# Predicting the potential distribution of the endangered red panda across its entire range using MaxEnt modeling

**DOI:** 10.1002/ece3.4526

**Published:** 2018-10-12

**Authors:** Arjun Thapa, Ruidong Wu, Yibo Hu, Yonggang Nie, Paras B. Singh, Janak R. Khatiwada, Li Yan, Xiaodong Gu, Fuwen Wei

**Affiliations:** ^1^ Key Lab of Animal Ecology and Conservation Biology Institute of Zoology Chinese Academy of Sciences Chaoyang, Beijing China; ^2^ International College University of Chinese Academy of Science Beijing China; ^3^ Institute of International Rivers and Eco‐Security Yunnan University Kunming Yunnan China; ^4^ Chengdu Institute of Biology Chinese Academy of Science Chengdu Sichuan China; ^5^ Sichuan Forestry Department Wildlife Conservation Division Chengdu Sichuan China

**Keywords:** habitat, Himalaya, predictive model, red panda

## Abstract

An upsurge in anthropogenic impacts has hastened the decline of the red panda (*Ailurus fulgens*). The red panda is a global conservation icon, but holistic conservation management has been hampered by research being restricted to certain locations and population clusters. Building a comprehensive potential habitat map for the red panda is imperative to advance the conservation effort and ensure coordinated management across international boundaries. Here, we use occurrence records of both subspecies of red pandas from across their entire range to build a habitat model using the maximum entropy algorithm (MaxEnt 3.3.3k) and the least correlated bioclimatic variables. We found that the subspecies have separate climatic spaces dominated by temperature‐associated variables in the eastern geographic distribution limit and precipitation‐associated variables in the western distribution limit. Annual precipitation (BIO12) and maximum temperature in the warmest months (BIO5) were major predictors of habitat suitability for *A. f. fulgens* and *A. f. styani*, respectively. Our model predicted 134,975 km^2^ of red panda habitat based on 10 percentile thresholds in China (62% of total predicted habitat), Nepal (15%), Myanmar (9%), Bhutan (9%), and India (5%). Existing protected areas (PAs) encompass 28% of red panda habitat, meaning the PA network is currently insufficient and alternative conservation mechanisms are needed to protect the habitat. Bhutan's PAs provide good coverage for the red panda habitat. Furthermore, large areas of habitat were predicted in cross‐broader areas, and transboundary conservation will be necessary.

## 1 INTRODUCTION

The red panda (*Ailurus fulgens*), an arboreal herbivorous mammal, occupies a highly specialized niche in which it dwells primarily in bamboo understories in temperate conifer forests adjacent to broadleaf forests (Wei, Feng, Wang, & Hu, 1999; Wei, Feng, Wang, Zhou, & Hu, 1999; Yonzon & Hunter, 1991). The red panda is a member of the order Carnivora but eats an herbivorous diet, particularly bamboo in humid climates at high altitudes (Wei, Feng, Wang, & Hu, 1999; Yonzon & Hunter, 1991). Presently, the red panda is distributed in five Asian countries: Nepal, India (Sikkim, West Bengal, Arunachala), Bhutan, northern Myanmar, and China (Tibet, Yunnan, and Sichuan) (Choudhury, 2001; Dorji, Rajaratnam, & Vernes, 2012; Glatston, 1994; Wei, Feng, Wang, & Hu, 1999; Yonzon & Hunter, 1991). It also may occurs a separate population in Meghalaya, India (Choudhury, 2001; Thapa, Hu, & Wei, 2018). Glatston (1994) and Wei, Feng, Wang, and Hu (1999) reported two subspecies (*A. fulgens fulgens* and *A. fulgens styani*) of red panda based on morphology and the geographic barrier of the Nujiang River in Yunnan, China. *A. f. fulgens* is regarded as the Himalayan subspecies confined to Nepal, India, Bhutan, Myanmar, and a small portion of Yunnan in China, whereas *A. f. styani* is known as the Chinese subspecies and is distributed in Sichuan and a part of Yunnan Province (Glatston, 1994; Wei, Feng, Wang, & Hu, 1999).

Despite its wide geographic range across the Himalayas, red panda is distributed patchily and occurs at low densities (Thapa et al., 2018; Wei, Feng, Wang, & Hu, 1999; Yonzon & Hunter, 1991). Habitat loss, fragmentation, and degradation are major threats to wild red pandas (Pradhan, Saha, & Khan, 2001; Wei, Feng, Wang, & Hu, 1999; Yonzon & Hunter, 1991). These factors have accelerated declines in wild populations, and the species is listed as endangered by the IUCN (Glatston, Wei, Than, & Sherpa, 2015). Likewise, occurring in a remote part of the Himalayan landscape, the red panda species remains poorly studied, and available database of the total species population is likely an underestimate due to scant occurrence records. Most studies to date have relied on observational surveys of indirect signs such as feces and pugmarks (Pradhan et al., 2001; Wei, Feng, Wang, & Hu, 1999; Yonzon & Hunter, 1991) as well as consultations with experts and local communities in small units (Jnawali, Leus, Molur, Glatston, & Walker, 2012; Wei, Traylor‐Holzer, Leus, & Glatston, 2014). This work has yielded qualitative information only and may not be representative of the entire geographic range. In addition, logistical constraints associated with frequent surveying of the often elusive and remote red panda explain occurrence data paucity (Kandel et al., 2015; Wei, Feng, Wang, & Hu, 1999). A comprehensive potential habitat map for the red panda across its entire range remains a gap in our understanding of this species and hampers effective, integrated, and holistic conservation. The species distribution model (SDM) is an appropriate solution to such challenges that can overcome sampling problems and generate reliable consistent and transparently derived estimate over large areas (Drew, Wiersma, & Huettmann, 2010) and that is appropriate to a species with a narrow ecological range (Hernandez, Graham, Master, & Albert, 2006) such as the red panda.

Climate plays an important role in determining species’ distributions, and evaluating the influence of climatic variables across a large geographic area (Morelle & Lejeune, 2015) to provide information about suitable habitat for a given species. Climatic variables are the dominant driving factors as opposed to distal variables such as elevation and topography, which are used frequently but have a low predictive performance (Bradie & Leung, 2017) . In an earlier study, Yonzon, Yonzon, Chaudhary, and Vaidya (1997) built a potential habitat model incorporating annual precipitation that was presumed as a good red panda (*A. f. fulgens*) distribution model in the Himalayas. In addition, temperature‐associated variables have contributed greatly to predicting red panda habitat in the vast Hindu Kush Himalaya region (Kandel et al., 2015). Additionally, temperature is a greater influencing factor than precipitation when building giant panda habitat models (Liu, Guan, Dai, Li, & Gong, 2016), where both the giant panda and the red panda (*A. f. styani*.) are sympatric macrohabitat dwellers. With these understandings, building potential distributions separately for subspecies is biologically meaningful, a fact that is not incorporated in previous studies. Furthermore, potential distribution modeling of the red panda based on species presence records only from Nepal (Kandel et al., 2015; Mahato, 2010) resulted in biased estimates for the large portion of red panda habitat located in Sichuan, China (Choudhury, 2001; Hu et al., 2011; Wei, Feng, Wang, & Hu, 1999; Wei et al., 2014). Here, we asked how well bioclimatic variables and topographic features predict the red panda's potential distribution range by comparing spatially filtered occurrence records from the entire range and least correlated climatic variable sets including both red panda subspecies.

Species distribution models are statistical models that use observed species distributional record data to infer species ecological requirements and map their potential distribution (Austin, 2002) . SDMs relate species presence records to mainly environmental factors to predict the potential distribution of a species across an area of interest (Elith, Ferrier, Huettmann, & Leathwick, 2005; Elith et al., 2006; Guisan & Thuiller, 2005; Pearson, Dawson, & Liu, 2004). SDMs have been implemented in managing biological invasions (NAT, 2009), identifying and protecting critical habitats (Heinrichs, Bender, Gummer, & Schumaker, 2010), selecting and translocating reserves (Seki, 2011), and building global species distribution range maps by the IUCN (Cord & Rodder, 2011; Jimenez‐Valverde, 2012). The most frequently used top five SDMs include MaxEnt, random forest, boosted regress trees, generalized additive models, and multivariate adaptive regression spines, all of which have similar predictive performances (García‐Callejas & Araújo, 2016). In this study, we used the maximum entropy algorithm (MaxEnt) because it is among the high‐performing, highly popular SDMs that use widely available presence‐only data, even dealing powerfully with limited occurrence data and small sample sizes (Fourcade, Engler, Rodder, & Secondi, 2014; Merow, Smith, & Silander, 2013; Phillips, Anderson, & Schapire, 2006; Phillips & Dudik, 2008). In addition, MaxEnt requires only species presence data, and both continuous and categorical environmental data can be used as input variables.

This study's goals were to (a) predict potential distribution of the red panda habitat across the entire range, (b) determine relevant influencing bioclimatic variables, (c) evaluate habitat conservation within existing protected areas (PAs), and (d) recommend conservation priority areas for future effective conservation. These findings provide insight into red panda habitat protection at the national and regional levels.

## 2 MATERIAL AND METHODS

### 2.1 Occurrence data and environment variables

**Figure 1:**
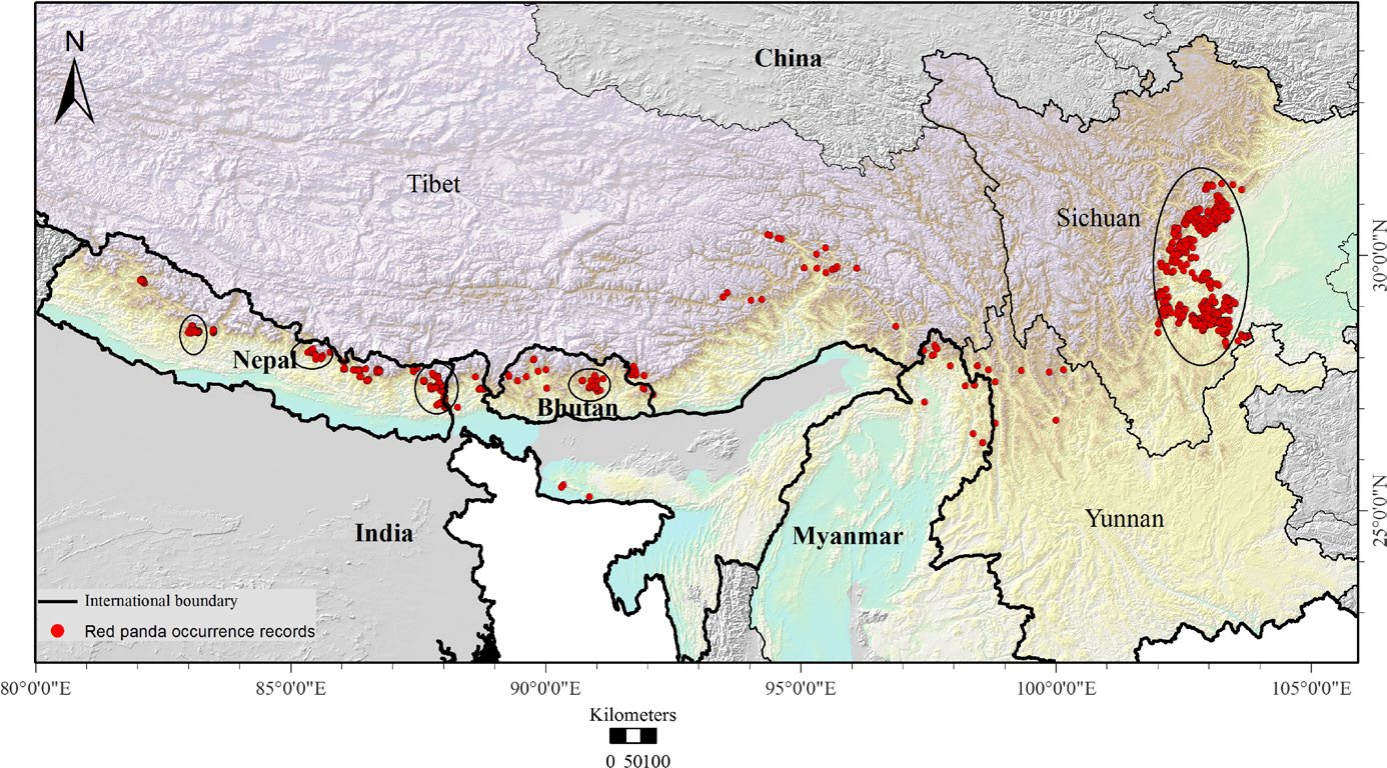
Occurrence records of red pandas in Nepal, India, Bhutan, Myanmar, and China. Circle indicates high clustered of occurrence records

We compiled red panda occurrence records from Nepal, India, Bhutan, Myanmar, and China (Supporting Information Table S1). Occurrence locations were based on presence data obtained from recent field studies (2015/2016 in Nepal), previous survey data (Nepal, China, and Bhutan), published work (Bhutan, India, Nepal, and Myanmar), museum specimen records (China and Nepal), and GBIF (http://www.gbif.org/). Feces, recognized by their distinct shape, was treated as the main indicator of red panda occurrence based on recommendations made by previous studies (Pradhan et al., 2001; Wei, Feng, Wang, & Hu, 1999; Yonzon & Hunter, 1991). Occurrence locations consisted of 3,050 presence records complied from distribution ranges in Nepal, India, Bhutan, Myanmar, and China to capture the westernmost and easternmost biogeographic distribution limits of the target species (Figure 1, Supporting Information Table S1). To model potential distribution, 19 bioclimatic raster layers were obtained from WorldClim (http://www.worldclim.com) which were 30 arc sec 9 (~1 km) in spatial resolution (Hijmans, Cameron, Parra, Jones, & Jarvis, 2005) (Supporting Information Table S2). These climatic layers represent annual trends (mean annual temperature and precipitation), seasonality (annual range in temperatures and precipitation), and limiting environmental factors (temperature and precipitation of a certain quarter) (Hijmans et al., 2005). Additionally, we derived aspect and slope from elevation data of WorldClim, which have similar resolution with climate variables.

### 2.2 Potential habitat prediction

To minimize collinearity among predictors, the variance inflation factor (VIF) was used under R v 2.15.0 (R Development Core Team, 2012); this factor has been implemented in many SDMs (Lauria, Gristina, Attrill, Fiorentino, & Garofalo, 2015; Ranjitkar, Xu, Shrestha, & Kindt, 2014; Ranjitkar, Kindt, et al., 2014). Here, we removed VIFs >10 (Supporting Information Tables S4 and S5) because strong collinearity affects model performance (Quinn & Keough, 2002). Finally, 10 statistical and biologically meaningful variables were used to model habitat distribution for the red panda (Supporting Information Tables S3, S4, and S9). To minimize the effect of spatial sampling bias (Su, Aryal, Nan, & Ji, 2015), we built spatial filters of grid sizes 2.5 km × 2.5 km (target animal's mean home range), 5 km × 5 km (Boria, Olson, Goodman, & Anderson, 2014), and 10 km × 10 km (Mainali et al., 2015), found to improve predictive performance in other studies. Our approaches included without spatial filter and with spatial filters of 2.5 km × 2.5 km and 5 km × 5 km grids from single occurrence locations extracted randomly from each grid (Supporting Information Table S1).

We employed the maximum entropy algorithm (MaxEnt 3.3.3k), one of the most robust and superior bioclimatic modeling approaches for presence‐only data (Elith et al., 2006, 2011; Wisz, Tamstorf, Madsen, & Jespersen, 2008) to map potential red panda habitat. MaxEnt has a few limitations that have been well discussed elsewhere, such as sampling bias of occurrence, the region used for background sampling, selection of features, and manipulation of the regularization multiplier (Elith et al., 2011; Kramer‐Schadt et al., 2013; Radosavljevic & Anderson, 2014). Our occurrence data (presence only) fit with MaxEnt (Elith et al., 2006; Phillips et al., 2006). Occurrence data collected in the field may possess sampling biases (e.g., numerous points near roads) that influence model performance due to increases in the spatial autocorrelation of localities (Boria et al., 2014). To control for potential bias, we used the spatial filter grids of different sizes mentioned above. We used mostly default settings in MaxEnt, except for the following settings: random test percentage equals 25% with 10‐fold cross‐validation and varying the values of the regularization multiplier. We manipulated the regularization multiplier values setting to 0.5, 1, 2, 3, 4, and 5 following recommendations (Anderson & Gonzalez, 2011; Aryal et al., 2016; Muscarella et al., 2014; Radosavljevic & Anderson, 2014; Su et al., 2015). We averaged the results of multiple runs from different models using three scenarios and six regularization multipliers. We selected linear, quadratic, and hinge features to avoid overfitting (Merow et al., 2013; Phillips & Dudik, 2008). Area under the curve (AUC) of the receiving operating curve was used to evaluate the accuracy of the model. AUC values range from 0 to 1 where the AUC values >0.5 show the model to be better than the randomly generated model (Phillips et al., 2006).

### 2.3 Model selection and validation

Akaike's information criteria (AIC_c_) (Burnham & Anderson, 2002) have outperformed all others, even when other methods corrected for small sample sizes (Warren & Seifert, 2011). Here, AIC_c_ values were used to determine the best fit models with the lowest values in ENMTools (Warren, Glor, & Turelli, 2010) using MaxEnt output files. The 10th percentile training presence as the suitability threshold was applied to build a potential suitability map, and the jackknife procedure was used to evaluate each predictor's relative importance. We imported reclassified data into three classes of habitat suitability, low (0.22–0.50 probability of occurrence), moderate (0.50–0.75 probability of occurrence), and high (<0.75 probability of occurrence), by omitting the values below the threshold as unsuitable habitat (Shrestha & Bawa, 2014). We applied FRAGSTATS (v 4.2) (McGarigal, Cushman, & Ene, 2012) to calculate the number of patches, mean patch size, and fragmental index in different countries.

We used two approaches to validate our model. First, expert opinion based on the Population and Habitat Viability Analysis database of Nepal and China (Jnawali et al., 2012; Wei et al., 2014) was used to cross‐validate whether confirmed districts/counties were predicted accurately by the model (Supporting Information Figure S4). Second, we validated the accuracy using the red panda presence databases of China (Biodiversity Profile Database of China) and Nepal (Database in Alaska Institutional Repository; https://scholarworks.alaska.edu/handle/11122/1012). Accuracy of the habitat suitability model was tested using the proportion of signs in each predicted habitat suitability probability index (HSPI) (higher the proportion of signs corresponded to higher accuracy). We used reclassified suitability maps (low, moderate, and high) in all countries to assess expert opinion and judgment (three red panda experts from each country) and cross‐validated these with our suitability maps.

### 2.4 Conservation assessment

We downloaded PA data for Nepal, India, Bhutan, and Myanmar from the World Database on Protected Areas (http://www.protectedplanet.net), and PA data for China from (Wu et al., 2011). To assess conservation status, we overlaid existing PAs with habitat suitability class and calculated the percentages of area included in PAs. Because red panda distribution is not equally partitioned among all countries, we calculated the proportion of the total area in each country; priority conservation areas were identified by overlaid raster layers of occurrence kernel density, predictive habitat suitability, and PAs using a raster calculator in ArcGIS. On the basis of the visual observation of the predicted map, we identified highly suitable habitat across international borders as habitat for transboundary conservation. Large areas of high suitability were identified as the most preferred for transboundary conservation.

## 3 RESULTS

### 3.1 Model selection, performance, and influencing variables

Of 72 candidate models, the best‐performing model for both subspecies was evaluated with the lowest AIC_c_ value (Supporting Information Tables S5 and S6). Eight variables for *A. f. fulgens* and seven for *A. f. styani* were not correlated (VIF > 10) and contributed significantly in the models. The lowest average AIC_c_ value (AIC_c_ = 4,762.66) was found for the 5 km × 5 km spatial filter and regularization parameter of 0.5 for *A. f. fulgens* representing Nepal, India, Bhutan, Myanmar, and Tibet (Supporting Information Table S5). For *A. f. styani*, a spatial filter of 5 km × 5 km and the regularization parameter of 1 had the lowest AIC_c_ (6,312.40) (Supporting Information Table S6), indicating that temperature‐associated variables predict habitat. The best models have a higher average training AUC (>0.97) and test AUC (>0.96), meaning they performed better than random when predicting habitat suitability of *A. fulgens*.

**Figure 2:**
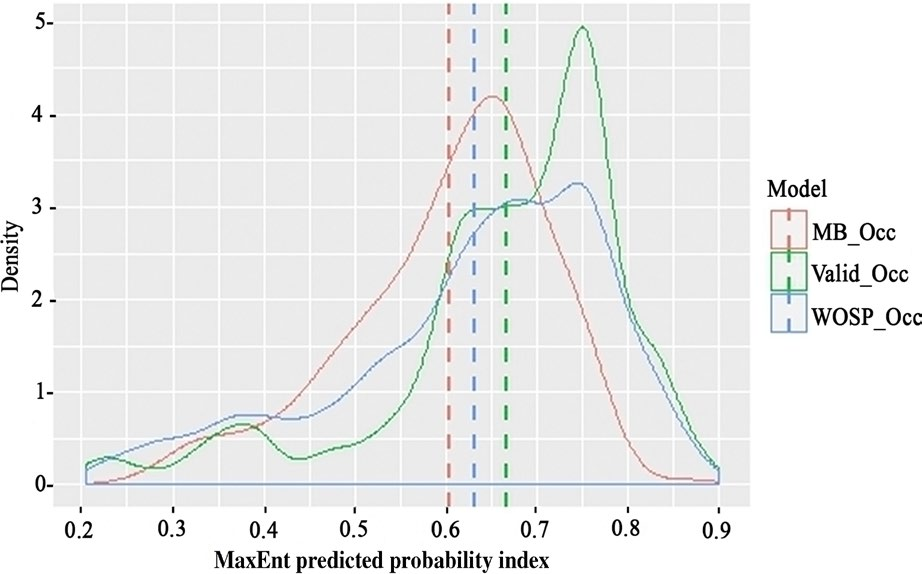
Density plot of the habitat suitability values of locations where red panda were recorded. MB_Occ is spatial filtered (5 km × 5 km) occurrence location used to build model. Valid_Occ is red panda occurrence information of red panda from Biodiversity Profile Database of China and open‐access database of Nepal (https://scholarworks.alaska.edu/handle/%2011122/1012). WOSP_Occ is red panda occurrence of red panda without spatial filtered. Dotted vertical lines indicate the mean predictive suitability for different occurrence data

The MaxEnt predictive probability index (0.22‐1 based on 10 percentile threshold) has an average suitability value above 0.6 in the density plot (Figure 2). A combination of the Biodiversity Profile China database and occurrence database from the Alaska repository found that 76% of occurrence location records had a habitat suitability probability above 0.5 according to the frequency distribution (Supporting Information Figure S9). This result indicates that our model has high precision and supports further model validation. The highly suitable habitat class encompassed 42.4% of occurrence records, followed by moderately (33.3% of records) and less suitable habitat (9.5% of records).

**Figure 3:**
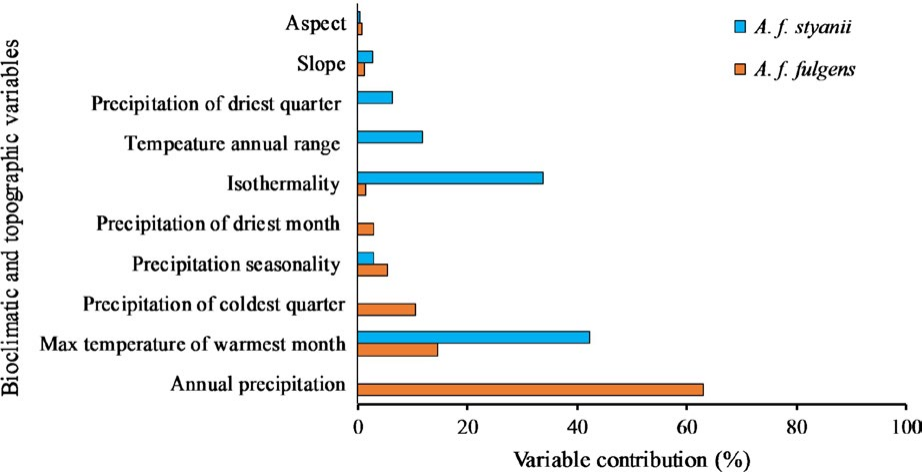
Relative importance of predictor variables in the predicted distributions of red panda subspecies

Temperature‐associated variables highly influenced habitat prediction for *A. f. styani*. Precipitation‐associated variables were predictive for *A. f. fulgens*. Annual precipitation was the most important variable in the *A. f. fulgens* model that contributed 63.1% in the model followed by maximum temperature in the warmest months (BIO 5), precipitation in the coldest quarter (BIO 19), precipitation seasonality (BIO 15), and precipitation in the driest month (BIO 14), accounting for relative gain contributions of 14.5%, 10.6%, 5.4%, and 2.9%, respectively (Figure 3). The relative contribution of three variables (BIO5, BIO3, and BIO7) accounted for more than 85% of habitat suitability prediction for *A. f. styani* in the model represented in the mountain ranges of Sichuan and Yunnan. The maximum temperature of the warmest month had the highest contribution (42.5%) to the model; followed by isothermality (33.0%), temperature annual range (11.7%), and precipitation in the driest quarter (6.3%) (Figure 3). Overall, climatic variables had a greater contribution than topographic variables (slope and aspect).

### 3.2 Predictive habitat suitability patterns

**Figure 4:**
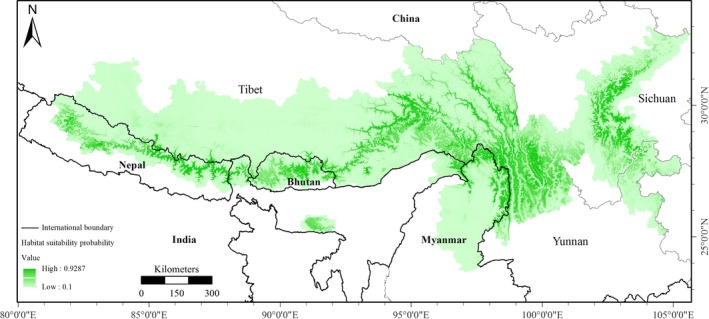
Predictive potential distribution map

**Figure 5:**
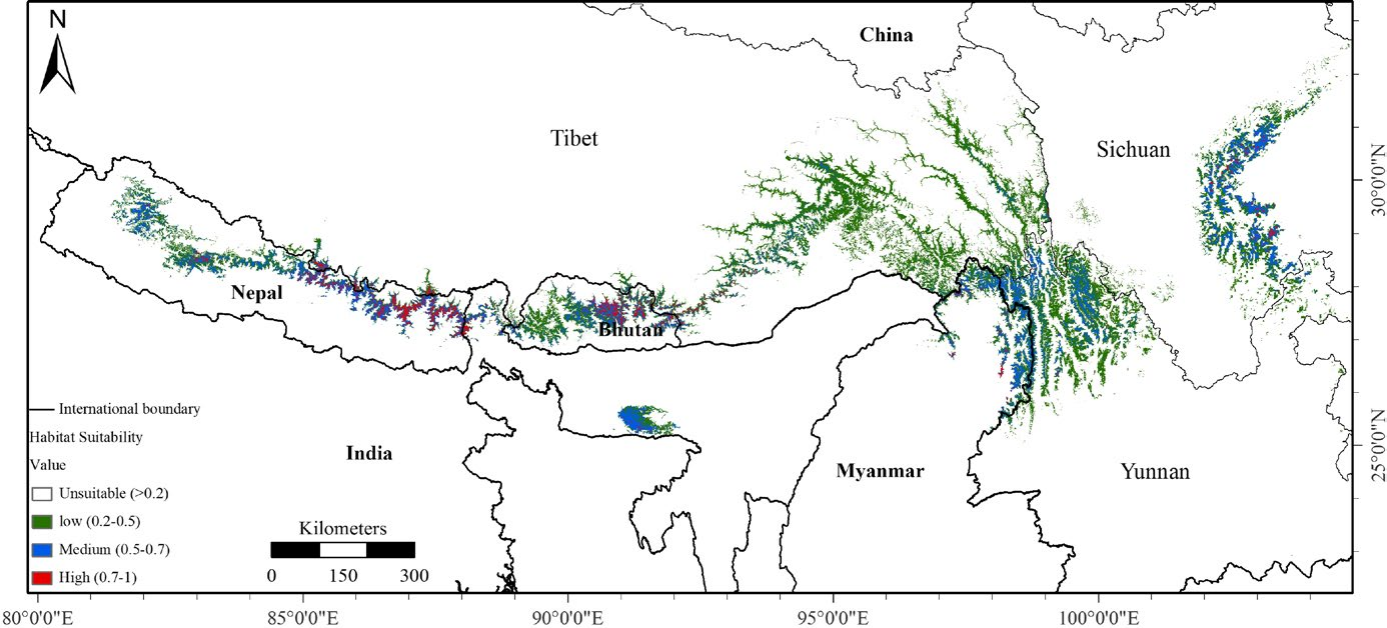
Habitat suitability of red panda based on predictive probability habitat class

Global red panda habitat is predicted to cover 134,975 km^2^ across China, Myanmar, Bhutan, India, and Nepal (Table 1 and Figure 4). China has a large amount of red panda habitat (61.24% of total habitat) compared to other countries (50% base on ≥0.5 probability), followed by Nepal, Myanmar, Bhutan, and India (Table 1). The HSPI aggregated mostly in high‐suitability and moderate‐suitability habitat (~above 50%) and not low‐suitability categories in all countries except China, indicating a large area of potential red panda habitat (Table 2). The western biogeographic distribution limit of Nepal has 20,150 km^2^ (14.93%) of predicted habitat, representing 13.69% of the total area of Nepal. Myanmar and Bhutan contain almost equal portions (~9%) of habitat, and India has only 5.29% of global red panda habitat. Our model predicted that 40% of habitat is highly or moderately suitable and 60% is low‐suitability habitat. Based on habitat classes in each country, moderately and highly suitable habitat comprises a significant proportion of suitable habitat in India (56.80%), Nepal (56.68%), and Myanmar (54.14%) but slightly low in Bhutan (45.24%) and China (32.30%) (Table 2 and Figure 5). Landscape matrix analysis showed that Tibet has a high number of patches, whereas Meghalaya has few patches, and Nepal and Sichuan have an equal number of patches (Supporting Information Table S7 and Figure S1). Sichuan, Yunnan, and Meghalaya have a low fragmentation index compared to Nepal, Bhutan, Myanmar, Sikkim, and Tibet. Highly and moderately suitable habitat classes have less fragmentation than poorly suitable habitat (Supporting Information Table S8 and Figure S3).

**Table 1:** Predicted red panda habitat across its entire range. The prediction is based on probability above 0.22 (10 percentile logistic threshold) and above 0.5 (core suitable habitat) and prediction within forest cover (land cover data of ICIMOD and land cover data of China)

Country	Habitat >0.22 probability (10 percentile threshold) (km^2^, %)	Habitat >0.5 probability (km^2^, %)	Habitat within forest (km^2^, %)
China	82,653 (61.24)	26,703 (49.90)	63,005 (76.23)
India	7,142 (5.29)	2,939 (5.49)	6,529 (91.41)
Nepal	20,150 (14.93)	5,614 (10.49)	15,721 (78.02)
Bhutan	12,407 (9.19)	6,835 (12.77)	12,171 (98.10)
Myanmar	12,623 (9.35)	11,422 (21.34)	9,195 (72.84)
Total	134,975 (100)	53,513 (100)	

**Table 2:** Habitat classes based on predictive probability range with area (km^2^, %) and distribution within each country

Habitat class	China	India	Nepal	Bhutan	Myanmar
Low suitability (0.22–0.5)	55,950 (67.69)	2,235 (43.19)	8,728 (43.31)	6,793 (54.75)	578 (49.85)
Moderate suitability (0.5–0.70)	23,774 (28.76)	2,531 (48.91)	8,541 (42.38)	4,086 (32.93)	6,207 (45.17)
High suitability (>0.70)	2,929 (3.54)	408 (7.88)	2,881 (14.29)	1,528 (12.31)	628 (4.97)

### 3.3 Conservation status and priority areas

Of the 134,975 km^2^ of predicted habitat, 37,711 km^2^ (27.93%) lie within 85 existing PAs in different IUCN categories (Table 3 and Supporting Information Figure S2). Overall, China contains 48.96% of red panda habitat, and other countries include 51% habitat inside PAs, was composed of 17.42%, 14.32%, 12.02%, and 7.29% for Nepal, Bhutan, Myanmar, and India, respectively. Across all PAs, Bhutan has the highest percentage of protected habitat, covering 43.52% (5,400 km^2^), followed by Myanmar (38.52%), Nepal (35.90%), India (32.60%), and China (22.33%). China's existing nature reserves (NRs) cover 18,459 km^2^ of habitat, accounting for 13.67% of the total predicted red panda habitat and 22.33% of the total NRs area, indicating a low percentage in comparison to other countries PAs (Table 3). PAs in Nepal and Bhutan include a large proportion of highly suitable habitat, whereas Myanmar, China, and Nepal have a large proportion of moderately suitable habitat. Current predicted habitat splits across management systems including national reserve, national park, wildlife sanctuary, hunting reserve, and biological corridor. China shares transboundary habitat with all countries including Tibet with Nepal, Bhutan, India, and Myanmar, and Yunnan with Myanmar (Supporting Information Figures S8 and S9). Moderate‐ and high‐suitability habitat predominately occur in central and eastern Nepal, central Bhutan, northern Myanmar, Sikkim (India), Yunnan, and Sichuan.

**Table 3:** Percent of predicted red panda habitat class inside protected area network in different countries

Country	Total area	Total area in protected areas	Protected (%)	Outside PAs	No. of PAs	PA coverage (%)	Habitat class under protected (%)		
							Low suitability	Moderate suitability	High suitability
China	82,653	18,459	22.33	77.67	31 PR, 16 NR, 2 CA	0.20	13.72	8.41	1.27
India	7,142	2,751	32.60	67.40	3 NP, 8 S	44.85	10.17	9.28	2.39
Nepal	20,150	6,569	35.90	64.10	5 NP, 4 CA, 1 HR	25.45	13.02	15.24	6.76
Bhutan	12,407	5,400	43.52	56.48	6 BC, 2 WS, 5 NP	31.90	24.12	17.24	7.04
Myanmar	12,623	4,532	38.52	61.48	2 WS, 1NP	16.18	16.97	18.93	2.13

Note

BC: biological corridor; CA: conservation area; HR: hunting reserve; NP: national park; NR: nature reserve; #PR: provincial; S: sanctuary; WS: wildlife sanctuary.

## 4 DISCUSSION

### 4.1 Influence of predictor variables

Climatic space for both subspecies identified by our modeling corresponds to the actual distribution of red pandas in the Himalayas and mountain ranges of Sichuan and Yunnan in China. However, actual habitat is likely smaller than predicted habitat because climatic variables are not the only determinants of red panda habitat suitability. Other factors such as edaphic and biogeographic factors limit the species distribution, even in areas that are climatically suitable (Ranjitkar, Kindt, et al., 2014). MaxEnt modeling approach has been implemented successfully to build current and future habitats under climate change scenarios for the sympatric macrohabitat dwellers red and giant panda (Li, Xu, Wong, Qiu, Li, et al., 2015; Li, Xu, Wong, Qiu, Sheng, et al., 2015; Liu et al., 2016; Songer, Delion, Biggs, & Huang, 2012; Sun, 2011). However, MaxEnt modeling has certain limitations, and recent studies suggest species‐specific tuning of the default manipulation improves model performance (Anderson & Gonzalez, 2011; Aryal et al., 2016; Radosavljevic & Anderson, 2014). Here, our model was corrected for sampling biases, calibrated with MaxEnt default settings, and model evaluation was based on robust evaluation statistics to overcome limitations. Use of spatial filtering reduces AIC_c_ values in models, increasing predictive performance and decreasing overfitting (Aryal et al., 2016; Boria et al., 2014). We adapted AIC as a model evaluation technique (Warren & Seifert, 2011), but solely using AUC has been criticized in many studies (Peterson, Papes, & Soberon, 2008). Our selected model has AUC values >0.9, which is recognized as an excellent model (Phillips et al., 2006). Our results and model trends remain consistent; importantly, 76% of the red panda occurrence records contained in the Biodiversity Profile Database of China and database of Alaska repository were overlaid within HSPI values above 0.5, indicative of high precision (Figure 2 and Supporting Information Figure S10).

One specific outcome in our model was to demonstrate whether environmental variables separately predict suitable habitat for each subspecies. Our results clearly show that the red panda subspecies can be separated in bioclimatic space in the Himalayas and mountain ranges of Yunnan and Sichuan (Supporting Information Figure S11). This result is consistent with Ranjitkar, Kindt, et al. (2014), who noted separate bioclimatic space in habitat suitability modeling of rhododendron trees in the Himalayas (Ranjitkar, Kindt et al., 2014), a major component of vegetation in red panda habitats. Temperature‐associated bioclimatic variables have great importance in predicting habitat suitability for *A. f. styani*, whereas precipitation‐associated bioclimatic variables were the most important habitat predictor for *A. f. fulgens*. Temperature and precipitation have a great influence over the growth rates of bamboo understories (Rao, Zhang, & Zhu, 1991), a primary food source for red pandas (Pradhan et al., 2001; Thapa & Basent, 2015; Wei, Feng, Wang, Zhou, et al., 1999; Yonzon, 1989; Zhang, Hu, Yang, Li, & Wei, 2009). High seasonality occurs in the eastern Himalaya and Hengduan Mountain, with high precipitation received during summer monsoonal rainfall (Kattel, Yao, Yang, Gao, & Tian, 2015). This corresponds with higher humidity during the warmest months in summer. Mostly bamboo species prefer to grow in the mild‐ and high‐humid climate (Li, Xu, Wong, Qiu, Li, et al., 2015; Li, Xu, Wong, Qiu, Sheng, et al., 2015) which is influenced by the humidity (Hu, Xiang, & Zhu, 1997). The moist areas and high‐humid condition contribute to the growth of bamboo shoot in the habitat during monsoon season that provides food resources for panda. Temperature also has a larger impact on giant panda distribution than rainfall (Songer et al., 2012). We assume environmental variables that affect giant pandas may directly influence red pandas because both the sympatric species have similar macrohabitat and dietary requirements (Wei, Feng, Wang, Zhou, et al., 1999; Zhang et al., 2004). Temperature is essential to individual growth, development, and survival and is thought to be the principal factor limiting giant panda geographic range (Wang, Ye, Skidmore, & Toxopeus, 2010; Xu et al., 2006). Meteorological data show that the climate in giant and red panda habitat has become warmer and drier in recent decades (Wang, Fan, Liu, & Chen, 2010) and that this pattern will continue.

Our model identified a strong response of *A. f. fulgens* to precipitation‐associated bioclimatic variables in the Himalayas, particularly annual precipitation, precipitation in the coldest quarter, precipitation seasonality, and precipitation in the driest month. Annual precipitation may increase in most parts of the regions (Liu, Xu, Henderson, & Qi, 2015). High precipitation occurs during the monsoon seasons in the Himalayas, which plays an important role in the growth of bamboo shoots that contribute highly nutritious food during the red panda breeding season. Furthermore, high precipitation implies increased cloud cover, leading to a significant reduction in soil temperature, reduced radiation input and high cloud albedo (Takahashi, Tokumitsu, & Yasue, 2005) resulting in delayed soil warming in spring, reduced tree growth, and slow understory regeneration. Precipitation seasonality and precipitation in the coldest quarter were recognized as important environmental factors, limiting red panda distribution in the Himalayas.

The topographic factors of slope and aspect emerged as contributive factors in our habitat model. Slope is a key factor determining feeding adaptation strategies in both panda species (Zhang, Wei, Li, & Hu, 2006). Elevation may indirectly affect red panda distribution, and it has a direct effect on the climatic condition of the given areas. Our results suggest that temperature and precipitation largely predict habitat suitability at a landscape level and may have a key role in shaping vegetation composition of the habitat. Climate‐associated variables are typically considered the most important determinant of species occurrence (Pearson & Dawson, 2003), when the model is extended to a large geographic area, and it provides basic information on suitable habitat for the species. Climate change can cause substantial species range contractions and extinctions and lead to a disproportionate distribution of a species along ecological zones (Wilson, Gutierrez, Gutierrez, & Monserrat, 2007), a factor not incorporated in our modeling.

### 4.2 Habitat patterns

Our model of current red panda habitat suitability performed well with biologically and statistically meaningful environmental variables (Supporting Information Table S9). Our best model estimated 134,975‐km^2^ potential red panda habitat across the entire range based on 10 percentile threshold. Kandel et al. (2015) estimated potential red panda habitat at approximately 47,000 km^2^ in the Hindu Kush Himalaya, which included 47.5% of predicted habitat within Nepal and at least 1% in Bhutan. Our model predicted the largest portion of red panda habitat in China, with more than half of the total habitat (61%) occurring in three provinces (Sichuan, Yunnan, and Tibet). Kandel et al. (2015) estimated only 27% red panda habitat in China, which was less than half of our estimated area; 5.5%, 22.7%, and 22.7% less habitat was estimated by Wei et al. (2014), Wei, Feng, Wang, & Hu (1999), Wei, Feng, Wang, Zhou, et al. (1999), and Choudhury (2001), respectively.

China has the largest amount of red panda habitat globally, and previous studies may have underestimated red panda habitat (Choudhury, 2001; Kandel et al., 2015; Wei, Feng, Wang, & Hu 1999). Importantly, our model prediction is robust and more comprehensive than prior methods (Choudhury, 2001; Kandel et al., 2015; Mahato, 2010; Wei, Feng, Wang, & Hu, 1999; Wei et al., 2014; Yonzon, Jones, & Fox, 1991). Our analysis is based on a machine‐learning algorithm, species records from the entire distribution range, and a separate set of climatic variables for both subspecies, which were not incorporated in previous studies by Wei, Feng, Wang, and Hu (1999), Wei, Feng, Wang, Zhou, et al. (1999), Yonzon et al. (1991), Choudhury (2001), Kandel et al. (2015), and Mahato (2010). In addition, Kandel et al. (2015) used occurrence records from Nepal, which include the Himalayan subspecies *A. fulgens fulgens* only, and this fact raises questions about undersampling, while predicting habitat across the landscape consists of climatic patterns and heterogeneous topography. Additionally, the Kandel et al. (2015) distribution map showed that the predicted presence was distributed beyond its westernmost biogeographic distribution limit (the Mugu District in Nepal), including Pakistan and Afghanistan, where the red panda is absent in wild habitat. Numerous studies have recognized that the MaxEnt algorithm produces highly accurate predictions over a wide range of species and geographic regions (Elith et al., 2006; Hernandez et al., 2006). Climate is a better driving factor than elevation, suggesting that microclimates in the Himalayas make for a powerful driver of red panda ecological niche and distribution (Kandel et al., 2015). At a given landscape, precipitation, temperature and elevation are highly correlated (Hof, Jansson, & Nilsson, 2012), and two or more correlated environmental variables can significantly decrease SDM accuracy.

Wei, Feng, Wang, and Hu (1999) and Wei, Feng, Wang, Zhou, et al. (1999) estimated red panda habitat in Sichuan, Yunnan, and Tibet to be 17,228.3 km^2^, 10,634.1 km^2^, and 9,574.1 km^2^, respectively, but we predicted it in Sichuan, Yunnan, and Tibet to be 25,962 km^2^, 28,196 km^2^, and 28,495 km^2^ based on a 10 percentile threshold (Figure S7). More recent estimates of red panda habitat in Sichuan (68,000 km^2^), Yunnan (42,000 km^2^), and Tibet (43,000 km^2^) (Wei et al., 2014) are roughly double our estimates. Among the Wei, Feng, Wang, and Hu (1999), Wei, Feng, Wang, Zhou, et al. (1999), Choudhury (2001), and Wei et al. (2014) estimates, estimated habitat in China varied greatly although similar methodological approaches were implemented. This fact may be due to recently updated survey information (Supporting Information Figure S7). Estimates by Wei et al. (2014) and Choudhury (2001) relied on forest cover in counties based on expert opinion, which excluded other ecological variables and species occurrence. Our model predicted 41 counties in China; however, Wei et al. (2014) identified 58 counties, and that might be a reason for habitat estimation variation (Supporting Information Table S1).

Our model predicted 20,150 km^2^ of suitable red panda habitat in Nepal, accounting for 17% of predicted habitat and comprising 60% of moderate‐ and high‐suitability habitat. Our prediction identified Nepal as a central area for conservation of *A. f. fulgens* in the Himalayas, similarly suggested by Kandel et al. (2015). Kandel et al. (2015) and Mahato (2010) predicted 22,400 km^2^ and 20,397 km^2^, respectively; however, Choudhury (2001) may have underestimated it at only 8,200 km^2^. Yonzon et al. (1997) estimated only 912 km^2^ of suitable habitat for red pandas in Nepal, which recorded the occurrence of the red panda only in 11 occurrence districts and used three parameters: fir forest (*Abies spectabilis*), elevation (3,000–4,000 m), and annual precipitation (>2,000 mm). Recently, the red panda was confirmed in 21 districts in Nepal. For example, Dolkha, Ramechhap (Thapa, Thapa, & Poudel, 2013), Kalikot (Bhatta, Shah, Devkota, Paudel, & Panthi, 2014), and Jajarkot (Baral, 2014) have been recorded beyond PAs and with an elevation range as low as 2,200 m in Ilam (RPN, 2016a, 2016b) in eastern Nepal and 2,400 m in Singalila National Park in India (Pradhan et al., 2001). Recently, occurrence of the red panda was confirmed in Rasuwa, Nuwakot, Myagdi, Baglung, and Dhading districts in central Nepal (Bista et al., 2017), which was predicted in our model. Likewise, the model estimated 12,623 km^2^ (9.3%) of red panda habitat in northern Myanmar, which is close to the estimate of Kandel et al. (2015) but different from Choudhury (2001). Due to limited studies on the red panda in Myanmar, it is difficult to justify clearly Myanmar's habitat estimation. MaxEnt's result suggested 46% of predicted habitat under PAs, 16% in biological corridors, and 38% outside the PA system in Bhutan (Glatston et al., 2015). Our result showed 17 districts suitable for the red panda in Bhutan, similar with Dorji, Vernes, and Rajaratnam (2011), who found 11 confirmed presence districts.

### 4.3 Conservation status

Existing PAs cover 27.93% (37,711 km^2^) of the predicted red panda habitat. This means that 72.07% of habitat important to red panda conservation does not currently have any legal protection, indicating a high probability of risk under growing anthropogenic activity and climate change. This region has recently had 39% of the Hindu Kush Himalayas converted to a PA network across eight countries (including Afghanistan and Pakistan), which is significant when compared to the global target of 10% (Chettri, Shakya, Thapa, & Sharma, 2008). PAs have proven to be effective for the protection of species against ongoing human threats but many species may shift their distributions outside existing PAs under climate change scenarios (Alagador, Cerdeira, & Araujo, 2014; Araujo, Alagador, Cabeza, Nogues‐Bravo, & Thuiller, 2011).

Protected area networks are good at representing the red panda habitat in Bhutan where PAs cover 43.52% of the red panda habitat, and represent 32.98% of Bhutan's PA network. In a former study, MaxEnt modeling showed that 46% of predicted the red panda habitat was within PAs, 16% was in biological corridors, and 38% was outside the PA system in Bhutan (Glatston et al., 2015). China contains a large amount of red panda habitat, but only 22.33% of habitat is under legal protection. Our model predicted 46 PAs suitable as red panda habitat in agreement with Wei and Zhang's (2011) 47 PAs, but in contrast to Wei, Feng, Wang, and Hu, (1999) and Wei, Feng, Wang, Zhou, et al. (1999) 31 reserves protecting 42.4% of habitat.

Ten mountainous PAs harbored suitable red panda habitat in Nepal representing 35.90% of predicted habitat and 19.29% of Nepal's PA network, indicating insufficient protection. PAs were skewed toward high mountains in Nepal leading to misrepresentation of important ecosystems, ecoregions, habitat, and species whereby current PAs provide insufficient protection for geophysical and biological entities (Shrestha, Shrestha, Chaudhary, & Chaudhary, 2010). Occurrence data showed that species records were more frequent within PAs than outside PAs, indicating surveys were focused in PAs in Nepal despite the fact that 64% of the habitat lies beyond PAs where recent field survey recorded additional new occurrence locations (e.g., Bhojpur, Dolpa, Ilam, Jajarkot, Kalikot, Lamjung, Myagdi, and Rolpa districts in Nepal) (Bista et al., 2017).

Red panda habitat is well predicted in northern parts of Myanmar, particularly in Hkakaborazi, Hponkanrazi, Bumhpabum, and Hukawng Valley PAs, representing 16.18% inside PAs. Other studies have recorded red pandas in Hkakaborazi and Hponkanrazi PAs (Zaw et al., 2008). Our model has good prediction across border areas between Yunnan and Myanmar, from where Dollman (1932) has recorded occurrence previously and Pocock (1941) (as citied in Zaw et al., 2008). Suitable habitat for red pandas was predicted in 11 PAs in India including in Sikkim and West Bengal State, but Meghalaya's habitat remains outside PAs. Approximately 50% of habitat is outside in PAs in Sikkim, similar to other studies that suggest up to 60% of habitat lies outside PAs (Glatston et al., 2015).

### 4.4 Priority habitat areas

Basically, it is expected that the higher the predictive presence probability/suitability at a site, the higher the site is to species survival. If the goal is to explore new populations and records, the highly suitable areas predicted in Nyirong, Nyalam, and Dinggye between Nepal and Tibet are recommended to initiate field survey. Tibet contains good habitat for red pandas and should be a high priority because of limited field studies. We recommend these potential habitats to initiate transboundary conservation (Supporting Information Figure S8). The large areas of predicted habitat and high occurrence kernel densities in central and eastern Nepal and Sikkim in India make this a good target for establish transboundary conservation (Supporting Information Figure S8). We recommend field surveys in the following mountain ranges because they contain predicted habitat beyond PAs: Shangri La, Meili Snow Mountain, Chall Snow Mountain, Gaoligong Mountain, Baima Snow Mountain, and Biluo Snow Mountain in Yunnan (Supporting Information Figure S5). Last, genetic studies suggest that the Xiaoxiangling population has different genetic types to *A. f. styani* compared to other mountains (Hu et al., 2011), and our model predicted highly suitable habitat and high kernel densities in the area (Supporting Information Figure S6).

### 4.5 Implications for red panda conservation

Our results showed that red panda conservation could not rely on existing PAs. Conservation beyond existing PAs should now be a focus of international effort. Around 70% of potential habitat is outside PAs, suggesting a need for buffer zone areas, community conservation sites, transboundary conservation zones, wildlife corridors, and special conservation sites. Due to the international distribution of red pandas and degree of cross‐border habitat, we recommend urgent initiation of a multilateral red panda conservation platform to secure the remaining wild population.

## AUTHOR CONTRIBUTIONS

AT and FWW designed the study, conducted data analysis. AT carried out fieldwork. AT, RW, YBH, YGN, PBS, JRK, LY, and XDG collected the data. AT, YBH, and FWW wrote the manuscript. All authors edited the final version.

## DATA ACCESSIBILITY

Relevant sampling sites information and climate data will be deposited in data repositories and available through Dryad (http://datadryad.org/) after acceptance of the manuscript.
