# Supplementary material for: Predicting the potential distribution of the endangered red panda across its entire range using MaxEnt modeling
Source: Ecol Evol. 2018 Oct 12;8(21):10542–54. doi: 10.1002/ece3.4526 (PMC6238126; doi:10.1002/ece3.4526)
Supplement: Supplementary file 1 [file ECE3-8-10542-s001.doc]

**Predicting the potential distribution of the endangered red panda across its entire range using MaxEnt Modeling**

Arjun Thapa^1,2^, Ruidong Wu^3^, Yibo Hu^1^, Yonggang Nie^1^, Paras Bikram Sigh^1,2^, Janak Raj Khatiwada^2,4^, Li Yan^1^, Xiaodong Gu^5^, Fuwen Wei^1,*^

*^1^ Key Lab of Animal Ecology and Conservation Biology, Institute of Zoology, Chinese Academy of Sciences, 1-5 Beichenxi Road, Chaoyang, Beijing 100101, China.*

*^2^International College, University of Chinese Academy of Science, Beijing, China.*

*^3^Institute of International Rivers and Eco-Security, Yunnan University, Kuming, Yunnan 650091, China.*

*^4^Chengdu Institute of Biology, Chinese Academy of Science, Chengdu, Sichuan, China.*

*^5^Sichuan Forestry Department, Wildlife Conservation Division, 610081, Chengdu, Sichuan, China*

**Corresponding email: weifw@ioz.ac.cn*

**Appendix -1 (Table S1- S9)**

**Supplementary Tables (Table S1- 9)**

Table S1. Species occurrence locations, data sources, occurrence with specified spatial filter.

| Country | Location | No. Occurrence | Spatial filter (km^2^) | | | | Data owner | Specific locations |
| --- | --- | --- | --- | --- | --- | --- | --- | --- |
|  |  |  | **2.5x2.5** | **5x5** | **10x10** | **Sources** |  |  |
|  | Western | 825 | 21 | 17 | 12 | Field survey | This study | Rara National Park, Dhorpatan Hunting Reserve Mugu, Jaumla, Jajarkot, Kalikot, Maygdi |
| Nepal | Central | 309 | 21 | 18 | 11 | Field survey | This study | Langtang National Park, Garuishskar Conservation Area, Tato pani, Kalinchock, Listikot, |
|  | Eastern | 956 | 50 | 36 | 23 | Field survey | This study  Tulsi Subedi (Personal Comm.) | Kanchangjunga Conservation Area, Sagarmatha National Park, Makalu Barun National Park, Illam, Panther, Taepjung |
|  | Sikkim | 62 | 4 | 4 | 3 | Field survey | Sunita Pradhan (Personal Comm.,)  Arundan Choudhary (Personal Comm.  Sunita Khatiwada (Personal Comm.) | Singhalila National Park, |
| India | West Bengal | 6 | 6 | 4 | 2 | Field survey | Sunita Khatiwada (Personal Comm.), | Kyongnosla Neora valley |
|  | Arunachala Pradesh | 10 | 8 | 8 | 5 | Literature |  |  |
|  | Meghalaya | 5 | 2 | 2 | 2 | Literature |  | East Kashi Hills, West Khasi hill |
| Bhutan |  | 40 | 31 | 25 | 16 | Field survey, literatures | Pemba Dinup (Personal Comm.) | Thumshingla, Bumthang,Jime Dorji, Sakteng |
| Myanmar |  | 12 | 12 | 12 | 10 | literatures | ZAW et al.(2008) | Hkakaborazi.Putao, Khawbude, Hsawlaw |
|  | Tibet | 20 | 20 | 20 | 16 | Field survey | Fuwen Wei Personal Comm.),  Yibu Hu | Tibet |
| China | Yunnan | 8 | 8 | 8 | 8 | Field survey | Fuwen Wei Personal Comm.),  Yibu Hu | Yunnan |
|  | Sichuan | 797 | 460 | 262 | 141 | Field survey | Fuwen Wei Personal Comm.),  Yibu Hu Personal Comm.) | Sichuan |
| Total |  | 3050 | 643 | 416 | 249 |  |  |  |

Table S2. Bioclimatic variables selected for *A. f. fulgens* and *A. f. styani.*

| Bioclimatic Variables (Bio_1-Bio_19 | *A.f. fulgens* | *A. f. styani* | |
| --- | --- | --- | --- |
| Annual Mean Temperature[BIO1] | - | - |  |
| Mean Diurnal Range (Mean of monthly (max temp-min temp)) [BIO2] | - | - |  |
| Isothermality (BIO2/BIO7)*(100)[BIO3] | Yes | Yes |  |
| Temperature Seasonality (standard deviation*100) [BIO4] | - | - |  |
| Max Temperature of Warmest Month[BIO5] | Yes | Yes |  |
| Min Temperature of Coldest Month [BIO6] | - | - |  |
| Temperature Annual Range (BIO5-BIO6)[BIO7] | - | Yes |  |
| Mean Temperature of Wettest Quarter [BIO8] | - | - |  |
| Mean Temperature of Direst Quarter[BIO9] | - | - |  |
| Mean Temperature of Warmest Quarter[BIO10] | - | - |  |
| Mean Temperature of Coldest Quarter [BIO11] | - | - |  |
| Annual Precipitation[BIO12] | Yes | - |  |
| Precipitation of Wettest Month[BIO13] | - | - |  |
| Precipitation of Driest Month [BIO14] | Yes | - |  |
| Precipitation Seasonality (Coefficient of Variation)[BIO15] | Yes | Yes |  |
| Precipitation of Wettest Quarter[BIO16] | - | - |  |
| Precipitation of Driest Quarter [BIO17] | - | Yes |  |
| Precipitation of Warmest Quarter [BIO18] | - | - |  |
| Precipitation of Coldest Quarter[BIO19] | Yes | - |  |
| Topographic | - | - |  |
| Aspect[ASP] | Yes | Yes |  |
| Slope[SLP] | Yes | Yes |  |

Table S3. VIF analysis of *A. f. styani.*

| Variables | bio_11 | bio_8 |  | bio_18 | bio_1 | bio_9 | bio_2 | bio_13 | bio_4 | bio_14 | bio_19 | bio_12 | VIF <10 |
| --- | --- | --- | --- | --- | --- | --- | --- | --- | --- | --- | --- | --- | --- |
| bio_11 | 6221.27 |  |  |  |  |  |  |  |  |  |  |  |  |
| bio_8 | 3206.40 | 2722.81 |  |  |  |  |  |  |  |  |  |  |  |
| bio_18 | 1954.05 | 1942.35 |  | 1941.59 |  |  |  |  |  |  |  |  |  |
| bio_1 | 3000.23 | 2305.11 |  | 1395.08 | 1312.20 |  |  |  |  |  |  |  |  |
| bio_9 | 1623.96 | 1034.62 |  | 962.15 | 1312.20 | 634.42 |  |  |  |  |  |  |  |
| bio_2 | 371.41 | 370.84 |  | 370.43 | 366.37 | 363.03 | 357.71 |  |  |  |  |  |  |
| bio_13 | 141.14 | 139.84 |  | 139.01 | 135.62 | 126.15 | 122.65 | 122.02 |  |  |  |  |  |
| bio_4 | 542.20 | 309.87 |  | 229.33 | 227.81 | 227.42 | 188.40 | 104.42 | 99.51 |  |  |  |  |
| bio_14 | 115.50 | 95.00 |  | 85.05 | 270.46 | 77.13 | 65.64 | 60.34 | 56.40 | 56.37 |  |  |  |
| bio_19 | 139.51 | 123.49 |  | 108.85 | 106.79 | 103.45 | 68.98 | 54.18 | 54.17 | 50.37 | 22.21 |  |  |
| bio_12 | 1246.05 | 1232.25 |  | 1230.57 | 366.37 | 90.44 | 89.15 | 78.39 | 21.36 | 18.23 | 17.40 | 12.42 |  |
| bio_3 | 137.32 | 135.67 |  | 135.63 | 135.62 | 130.93 | 130.02 | 78.23 | 77.09 | 5.58 | 5.41 | 2.22 | 2.10 |
| bio_5 | 1548.50 | 1492.97 |  | 1333.04 | 1023.47 | 583.47 | 3.55 | 3.55 | 3.41 | 3.21 | 2.75 | 2.51 | 2.44 |
| bio_7 | 293.27 | 292.48 |  | 290.14 | 270.46 | 263.37 | 222.20 | 35.49 | 34.85 | 4.08 | 3.77 | 3.04 | 3.01 |
| bio_14 | 43.41 | 43.22 |  | 43.20 | 227.81 | 33.82 | 32.11 | 30.91 | 30.81 | 26.93 | 20.34 | 10.13 | 2.90 |
| bio_15 | 78.17 | 72.58 |  | 72.45 | 1023.47 | 28.80 | 28.60 | 26.77 | 10.84 | 10.70 | 10.70 | 4.28 | 3.29 |
| slope | 1.08 | 1.08 |  | 1.08 | 1.08 | 1.08 | 1.08 | 1.07 | 1.06 | 1.06 | 1.06 | 1.06 | 1.06 |
| aspect | 1.09 | 1.09 |  | 1.09 | 1.09 | 1.08 | 1.08 | 1.08 | 1.08 | 1.07 | 1.07 | 1.04 | 1.04 |

Table S4. VIF analysis of *A. f. fulgens*.

| Variables | bio_11 | bio_10 | bio_1 | bio_16 | bio_13 | bio_18 | bio_8 | bio_17 | bio_4 | bio_9 | VIF <10 |
| --- | --- | --- | --- | --- | --- | --- | --- | --- | --- | --- | --- |
| bio_11 |  |  |  |  |  |  |  |  |  |  |  |
| bio_10 | 4289.30 |  |  |  |  |  |  |  |  |  |  |
| bio_1 | 2890.57 | 1153.22 |  |  |  |  |  |  |  |  |  |
| bio_16 | 937.93 | 936.09 | 929.68 |  |  |  |  |  |  |  |  |
| bio_13 | 426.74 | 426.56 | 407.20 | 320.04 |  |  |  |  |  |  |  |
| bio_18 | 279.65 | 278.89 | 276.23 | 271.48 | 216.21 |  |  |  |  |  |  |
| bio_8 | 1388.90 | 777.91 | 238.92 | 230.85 | 209.83 | 153.76 |  |  |  |  |  |
| bio_17 | 229.40 | 217.29 | 195.99 | 170.22 | 158.08 | 154.79 | 154.71 |  |  |  |  |
| bio_4 | 217.24 | 172.35 | 120.29 | 119.82 | 95.09 | 88.89 | 73.55 | 73.55 |  |  |  |
| bio_9 | 143.08 | 139.75 | 131.46 | 125.91 | 103.84 | 103.33 | 69.40 | 68.23 | 57.81 |  |  |
| bio_2 | 509.31 | 509.30 | 506.67 | 493.80 | 86.56 | 81.75 | 71.19 | 66.31 | 37.87 | 4.45 |  |
| bio_3 | 27.23 | 27.06 | 27.06 | 26.82 | 12.68 | 12.58 | 11.24 | 11.23 | 5.30 | 3.65 | 1.88 |
| bio_5 | 176.61 | 166.02 | 123.86 | 123.36 | 104.18 | 75.43 | 38.95 | 37.43 | 29.69 | 2.79 | 2.02 |
| bio_12 | 717.65 | 713.84 | 686.15 | 273.10 | 154.35 | 11.68 | 8.48 | 8.33 | 8.05 | 7.48 | 4.06 |
| bio_14 | 31.64 | 31.57 | 22.14 | 21.26 | 20.82 | 20.81 | 16.51 | 5.66 | 5.65 | 4.75 | 4.30 |
| bio_15 | 27.60 | 27.58 | 25.66 | 22.66 | 19.28 | 7.73 | 7.23 | 6.85 | 5.49 | 5.42 | 3.48 |
| bio_19 | 151.41 | 147.06 | 134.36 | 128.50 | 118.52 | 100.51 | 77.70 | 10.68 | 8.80 | 7.34 | 2.93 |
| slp | 1.31 | 1.29 | 1.29 | 1.29 | 1.25 | 1.23 | 1.21 | 1.21 | 1.16 | 1.14 | 1.11 |
| aspect | 1.31 | 1.40 | 1.40 | 1.34 | 1.31 | 1.27 | 1.24 | 1.16 | 1.13 | 1.13 | 1.12 |

Table S5. Performance of MaxEnt models in predicting species distribution of *A. f. fulgens* (Himalayan subspecies).

| **Model**  **scenario** |  | **Bioclimatic + topography** | | | | |
| --- | --- | --- | --- | --- | --- | --- |
|  | Regularization  Multiplier | Mean AIC | Mean AICc | Mean BIC | Training AUC | Test AUC |
| **Normal** | 0.5 | 4766.64 | 5816.82 | 5192.77 | 0.97 | 0.95 |
|  | 1 | 4773.55 | 4822.97 | 4976.39 | 0.97 | 0.95 |
|  | 2 | 4731.98 | 4777.54 | 4922.67 | 0.95 | 0.95 |
|  | 3 | 4756.26 | 4785.07 | 4912.67 | 0.95 | 0.94 |
|  | 4 | 4761.73 | 4779.68 | 4888.52 | 0.95 | 0.94 |
|  | 5 | 4765.52 | 4776.71 | 4867.35 | 0.95 | 0.94 |
| **2.5 km × 2.5 km** | 0.5 | 4821.14 | 5046.08 | 5178.79 | 0.97 | 0.94 |
|  | 1 | 4835.00 | 4885.19 | 5037.79 | 0.96 | 0.94 |
|  | 2 | 4865.39 | 4882.79 | 4992.76 | 0.95 | 0.94 |
|  | 3 | 4879.39 | 4886.32 | 4962.52 | 0.95 | 0.94 |
|  | 4 | 4879.39 | 4886.32 | 4962.52 | 0.95 | 0.94 |
|  | 5 | 4909.42 | 4912.94 | 4969.08 | 0.94 | 0.94 |
| **5 km × 5 km** | 0.5 | 4762.66 | 5004.47 | 5132.40 | 0.97 | 0.95 |
|  | 1 | 4789.66 | 4846.6 | 5005.45 | 0.96 | 0.95 |
|  | 2 | 4831.96 | 4852.37 | 4970.11 | 0.96 | 0.95 |
|  | 3 | 4762.66 | 5004.47 | 5132.40 | 0.95 | 0.95 |
|  | 4 | 4848.83 | 4854.59 | 4925.13 | 0.95 | 0.95 |
|  | 5 | 4858.77 | 4862.72 | 4922.64 | 0.95 | 0.95 |

Table S6. Performance of MaxEnt models in predicting distribution of *A. f. styani* (Chinese sub species).

| Model  scenario | Regularization  Multiplier | Bioclimatic + topography | | | | |
| --- | --- | --- | --- | --- | --- | --- |
|  |  | Mean AIC | Mean AICc | Mean BIC | Training AUC | Test AUC |
| Normal | 0.5 | 18004.56 | 18031.81 | 18469.14 | 0.96 | 0.95 |
|  | 1 | 18040.1 | 18053.55 | 18372.28 | 0.96 | 0.95 |
|  | 2 | 18020.74 | 18026.24 | 18235.6 | 0.96 | 0.96 |
|  | 3 | 18083.69 | 18086.31 | 18232.58 | 0.96 | 0.95 |
|  | 4 | 18084.64 | 18087.23 | 18231.64 | 0.96 | 0.96 |
|  | 5 | 18130.49 | 18132.57 | 18263.36 | 0.96 | 0.96 |
| Spatial filter  2.5 km × 2.5 km | 0.5 | 10551.99 | 10602.6 | 10959.83 | 0.97 | 0.96 |
|  | 1 | 10561.88 | 10582.39 | 10831.92 | 0.97 | 0.96 |
|  | 2 | 10585.7 | 10595.09 | 10771.53 | 0.96 | 0.96 |
|  | 3 | 10619.81 | 10626.02 | 10772.23 | 0.96 | 0.96 |
|  | 4 | 10650.32 | 10655.23 | 10786.04 | 0.96 | 0.95 |
|  | 5 | 10685.61 | 10689.47 | 10806.31 | 0.96 | 0.96 |
| Spatial filter  5 km × 5 km | 0.5 | 6419.20 | 6634.52 | 6874.65 | 0.97 | 0.96 |
|  | 1 | 6340.12 | 6445.45 | 6695.57 | 0.97 | 0.96 |
|  | 2 | 6365.33 | 6372.78 | 6518.49 | 0.97 | 0.96 |
|  | 3 | 6378.96 | 6389.79 | 6509.34 | 0.97 | 0.96 |
|  | 4 | 6400.22 | 6407.35 | 6506.92 | 0.96 | 0.96 |
|  | 5 | 6519.20 | 6624.52 | 6874.65 | 0.96 | 0.96 |

Table S7. Landscape characteristics of red panda habitat (area unit: Km^2^).

| Country/States | Area | Mean patch size | Minimum  Area | No. of patch | Fragmentation Index |
| --- | --- | --- | --- | --- | --- |
| Meghalaya (India) | 3,503 | 27.89 | 0.01 | 241 | 0.065 |
| Sikkim (India) | 3,639 | 31.93 | 0.01 | 638 | 0.175 |
| Myanmar | 9,613 | 82.36 | 0.01 | 975 | 0.101 |
| Bhutan | 12,407 | 24.42 | 0.01 | 1248 | 0.101 |
| Yunnan (China) | 28,196 | 41.38 | 0.01 | 1954 | 0.069 |
| Nepal | 20,150 | 29.55 | 0.01 | 2454 | 0.122 |
| Sichuan (China) | 25,962 | 59.00 | 0.01 | 2498 | 0.096 |
| Tibet (China) | 28,495 | 122.25 | 0.01 | 3918 | 0.137 |

**Table S8.** Landscape characteristics of red panda habitat classes and locations (area Km^2^).

| Country/States | Habitat Class | Class Area (Km^2^) | Mean Patch Size | No. Patch | Patch Density | Large Patch Index |
| --- | --- | --- | --- | --- | --- | --- |
| Bhutan | Low | 6,793 | 12.57 | 540 | 0.043 | 0.1013 |
|  | Moderate | 4,086 | 8.90 | 459 | 0.036 | 0.071 |
|  | High | 1,528 | 6.13 | 249 | 0.028 | 0.049 |
| Sikkim, Arunachala, West Bengal (India) | Low | 634 | 3.84 | 165 | 0.098 | 0.229 |
|  | Moderate | 701 | 7.53 | 93 | 0.05 | 0.451 |
|  | High | 336 | 3.862 | 87 | 0.05 | 0.231 |
| Meghalaya (India) | Low | 1,601 | 14.04 | 114 | 0.03 | 0.400 |
|  | Moderate | 1,830 | 19.46 | 94 | 0.02 | 0.555 |
|  | High | 72 | 2.18 | 33 | 0.007 | 0.062 |
| Myanmar | Low | 4,420 | 7.281 | 607 | 0.063 | 0.075 |
|  | Moderate | 4,708 | 23.07 | 204 | 0.049 | 0.240 |
|  | High | 485 | 2.95 | 164 | 0.017 | 0.030 |
| Nepal | Low | 8,728 | 6.79 | 1,284 | 0.063 | 0.033 |
|  | Moderate | 8,541 | 10.34 | 826 | 0.040 | 0.051 |
|  | High | 2,881 | 8.37 | 344 | 0.017 | 0.041 |
| Sichuan | Low | 12,673 | 7.28 | 1,739 | 0.137 | 0.028 |
|  | Moderate | 11,716 | 24.56 | 477 | 0.018 | 0.094 |
|  | High | 1,573 | 5.57 | 282 | 0.010 | 0.021 |
| Tibet | Low | 23,191 | 10.57 | 2,194 | 0.076 | 0.037 |
|  | Moderate | 4101 | 3.11 | 1,317 | 0.046 | 0.010 |
|  | High | 1,203 | 2.95 | 407 | 0.014 | 0.010 |
| Yunnan | Low | 20,086 | 16.58 | 1,211 | 0.042 | 0.058 |
|  | Moderate | 7,957 | 12.29 | 647 | 0.022 | 0.043 |
|  | High | 153 | 1.593 | 96 | 0.003 | 0.005 |

Table S9. Variables to use to build the final model and contributions in model building

| Variables | Description | Contribution (%) | | Variables included in previous studies |
| --- | --- | --- | --- | --- |
|  |  | *A.f.fulgens* | *A.f.styani* |
| BIO 12 | Annual Precipitation | 63.1 | ─ | Yonzon et al., 1997; Kandel et al., 2016 (Red panda) |
| BIO 5 | Max Temperature of Warmest Month | 14.5 | 42.3 | Kandel et al. 2015 (Red panda) |
| BIO 19 | Precipitation of Coldest Quarter | 10.6 | ─ | Mahato, 2010; Kandel et al. 2015 (Red panda) |
| BIO 15 | Precipitation Seasonality (Coefficient of Variation) | 5.4 | 3 | Mahato, 2010; Kandel et al. 2015 (Red panda), Li et al. 2014 (Giant panda) |
| BIO 14 | Precipitation of Driest Month | 2.9 | ─ | Li et al. 2014 (Giant panda habitat) |
| BIO 3 | Isothermality (BIO2/BIO7)*(100) | 1.5 | 33.8 | Kandel et al. 2015 (Red panda) |
| BIO 7 | Temperature Annual Range (BIO5-BIO6) | ─ | 11.7 | Kandel et al. 2015 (Red panda) |
| BIO 17 | Precipitation of Driest Quarter | ─ | 6.3 | Kandel et al. 2015 (Red panda habitat model), Li et al. 2014 (Giant panda) |
| SLP | Slope | 1.2 | 2.6 | Yonzon et al. 1997 (Red panda); Li et al. 2014 (Giant panda) |
| ASP | Aspect | 0.8 | 0.3 | Yonzon et al.1997 (Red panda) |
