# Supplementary material for: Predicting the potential distribution of the endangered red panda across its entire range using MaxEnt modeling
Source: Ecol Evol. 2018 Oct 12;8(21):10542–54. doi: 10.1002/ece3.4526 (PMC6238126; doi:10.1002/ece3.4526)
Supplement: Supplementary file 2 [file ECE3-8-10542-s002.doc]

**Predicting the potential distribution of the endangered red panda across its entire range using MaxEnt Modeling**

Arjun Thapa^1,2^, Ruidong Wu^3^, Yibo, Hu^1^, Yonggang Nie^1^, Paras Bikram Sigh^1,2^, Janak Raj Khatiwada^2,4^, Li Yan^1^, Xiaodong Gu^5^, Fuwen Wei^1,*^

*^1^ Key Lab of Animal Ecology and Conservation Biology, Institute of Zoology, Chinese Academy of Sciences, 1-5 Beichenxi Road, Chaoyang, Beijing 100101, China.*

*^2^International College, University of Chinese Academy of Science, Beijing, China.*

*^3^Institute of International Rivers and Eco-Security, Yunnan University, Kuming, Yunnan 650091, China.*

*^4^Chengdu Institute of Biology, Chinese Academy of Science, Chengdu, Sichuan, China.*

*^5^Sichuan Forestry Department, Wildlife Conservation Division, 610081, Chengdu, Sichuan, China*

**Corresponding email: weifw@ioz.ac.cn*

**Appendix-2**

**Supplementary Figures (Fig.S1-S10)**


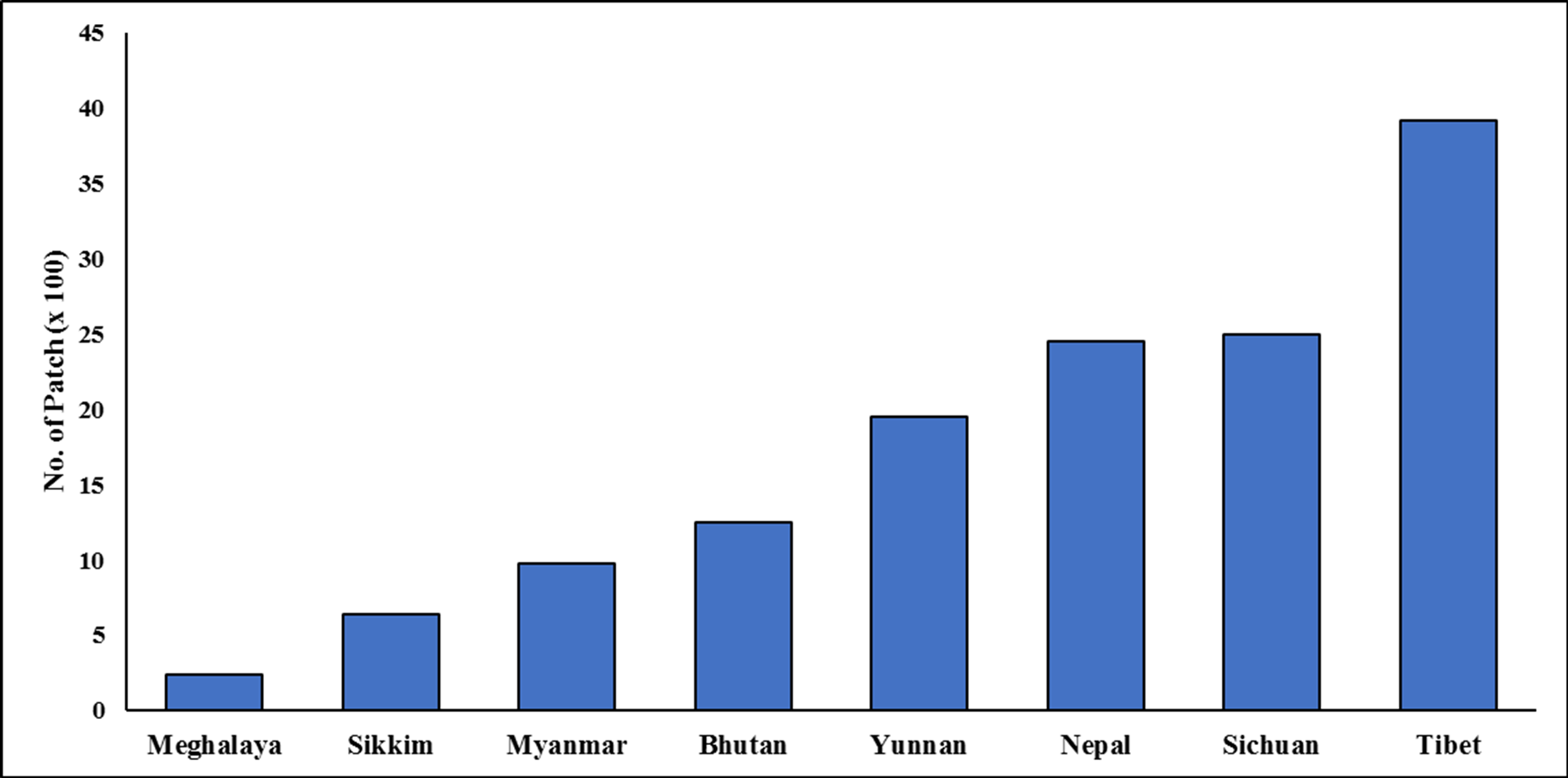


Fig. S 1. Number of habitat patches.


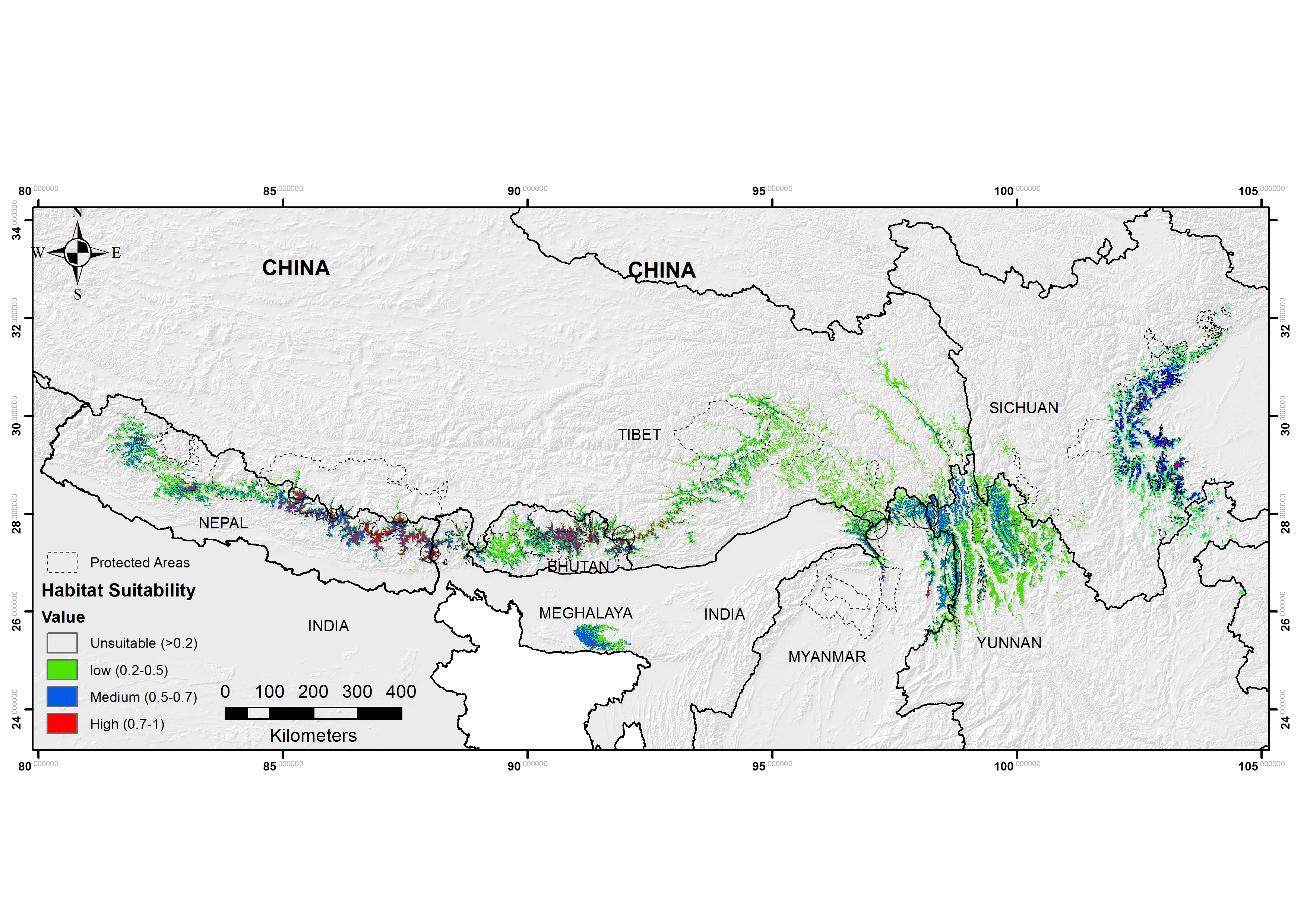


**Fig. S 2.** Predicted red panda habitat classes inside protected area networks.


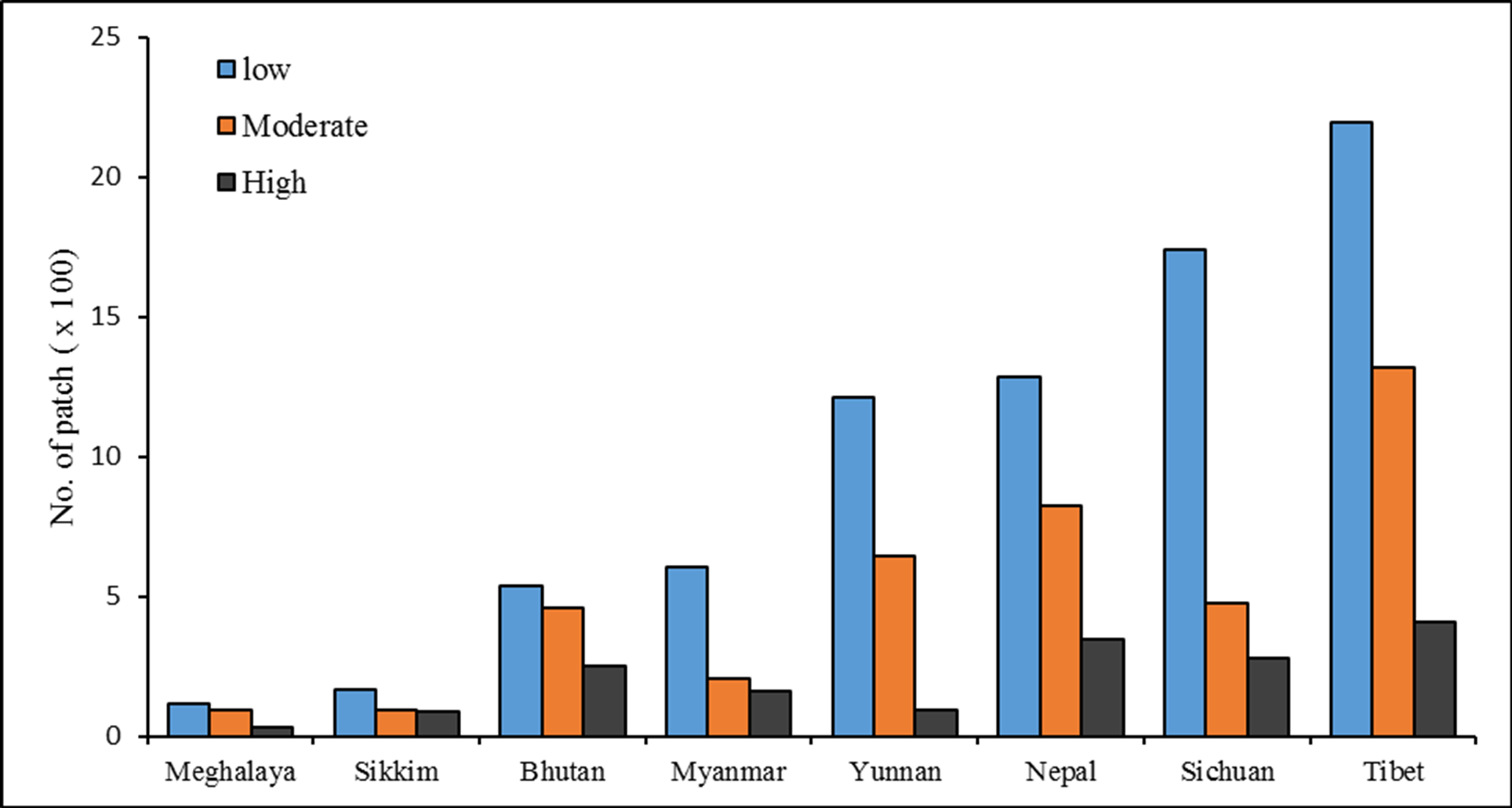


Fig. S3. Number of habitat patches and habitat classes.

**
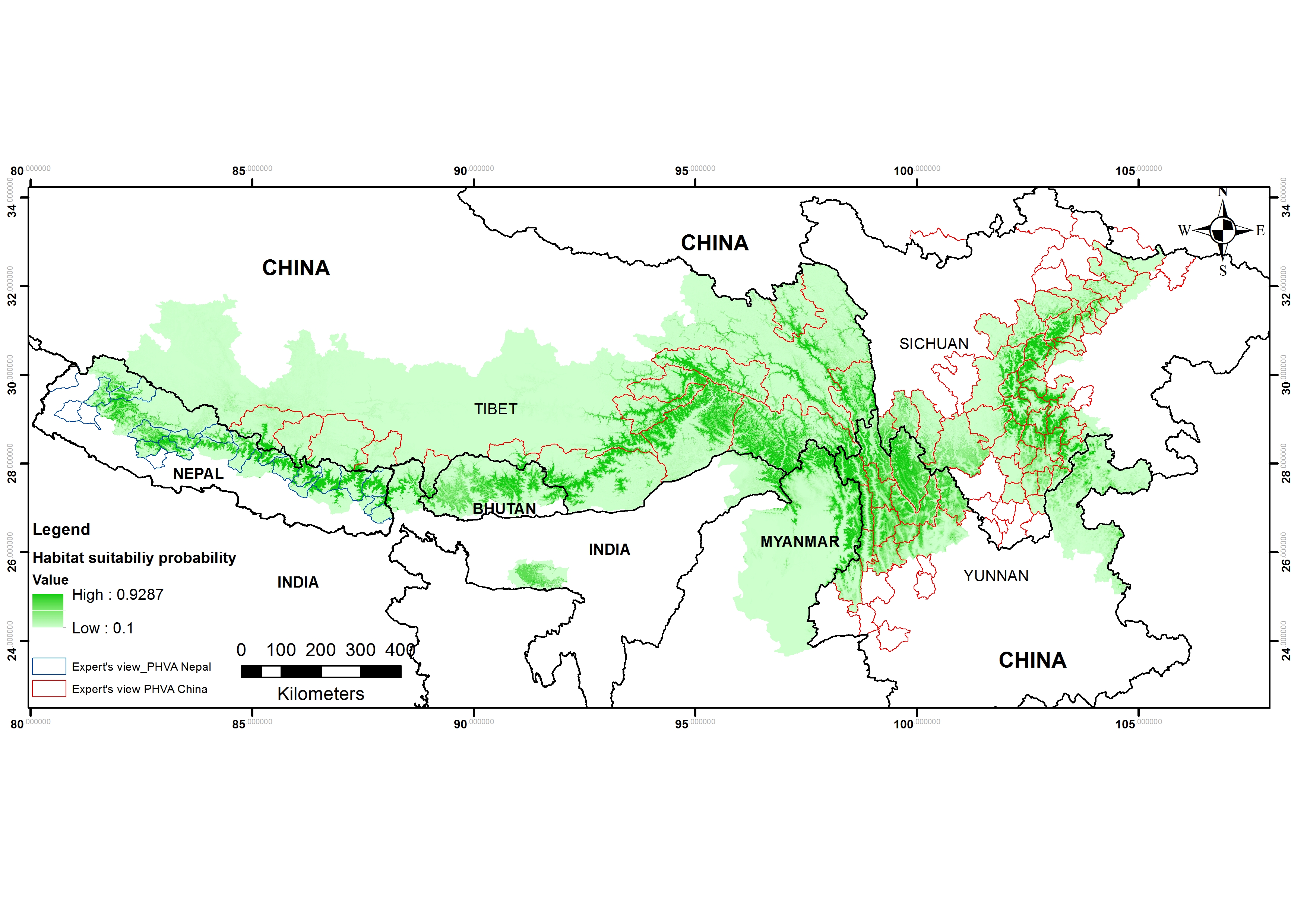
**

**Fig. S4.** Predictive habitat overlay using expert views (PHVA database of Nepal and China).


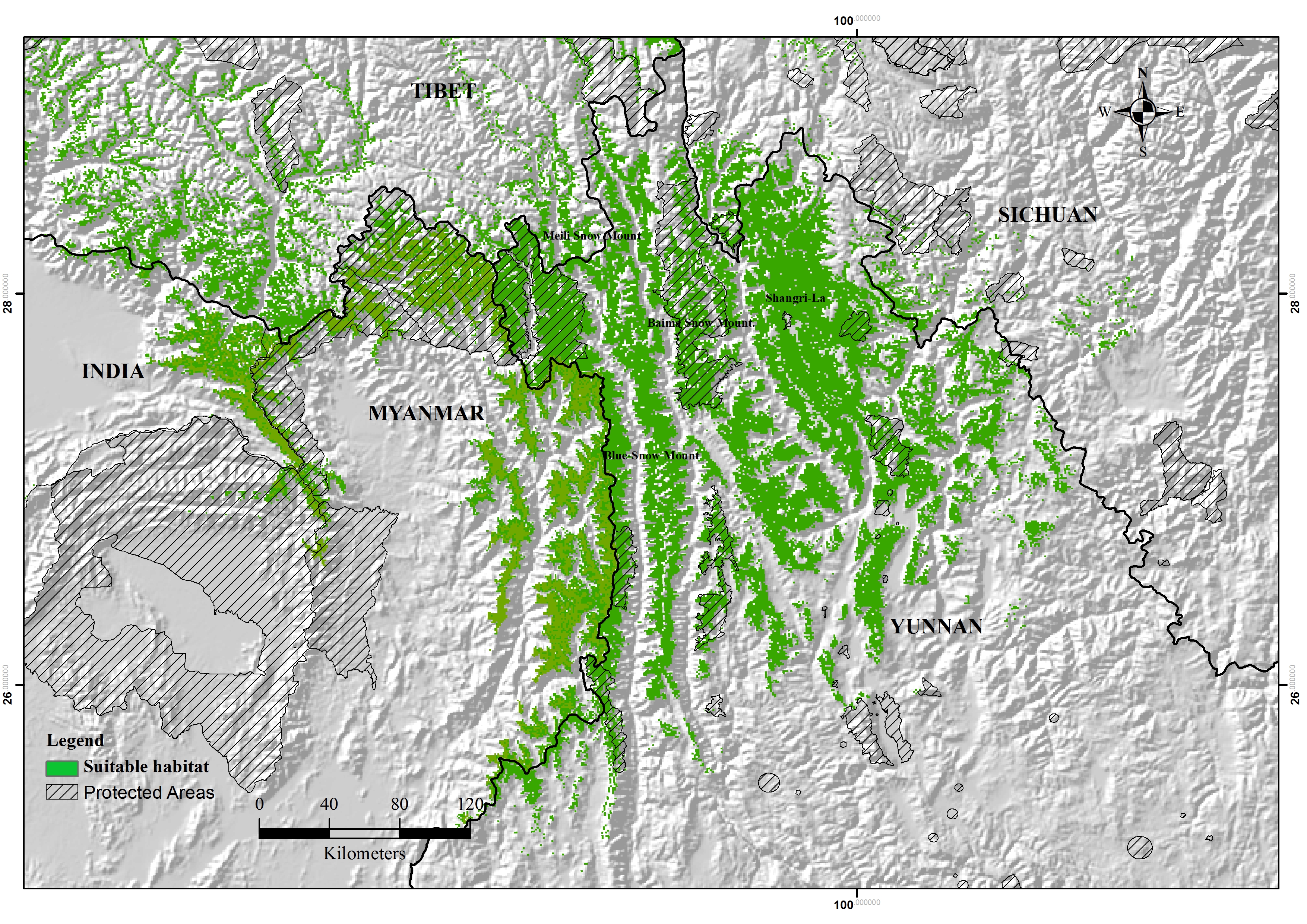


**Fig. S5.** Different Mountain ranges and red panda habitat in Yunnan.


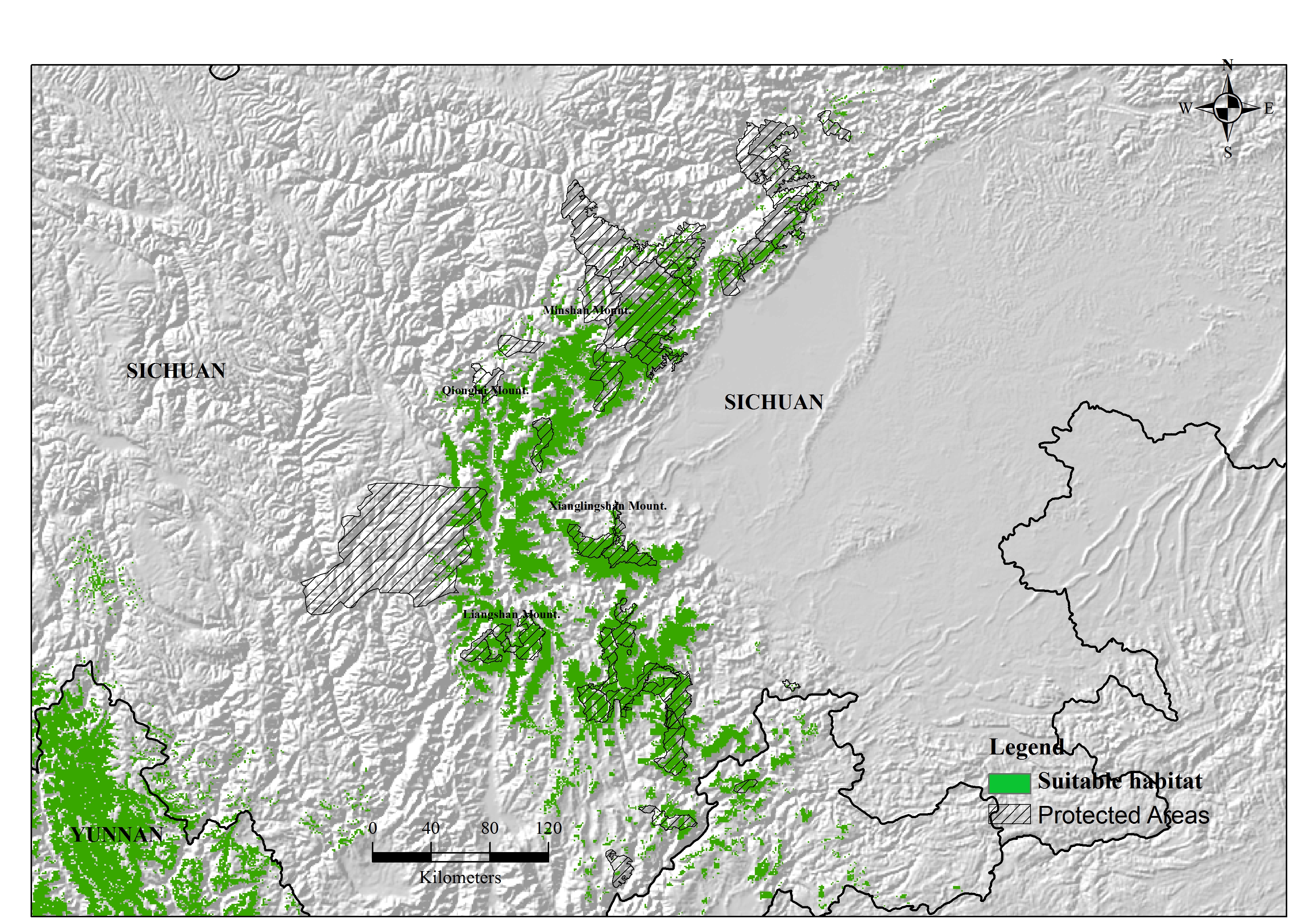


**Fig. S6**. Different Mountain ranges and red panda habitat in Sichuan.


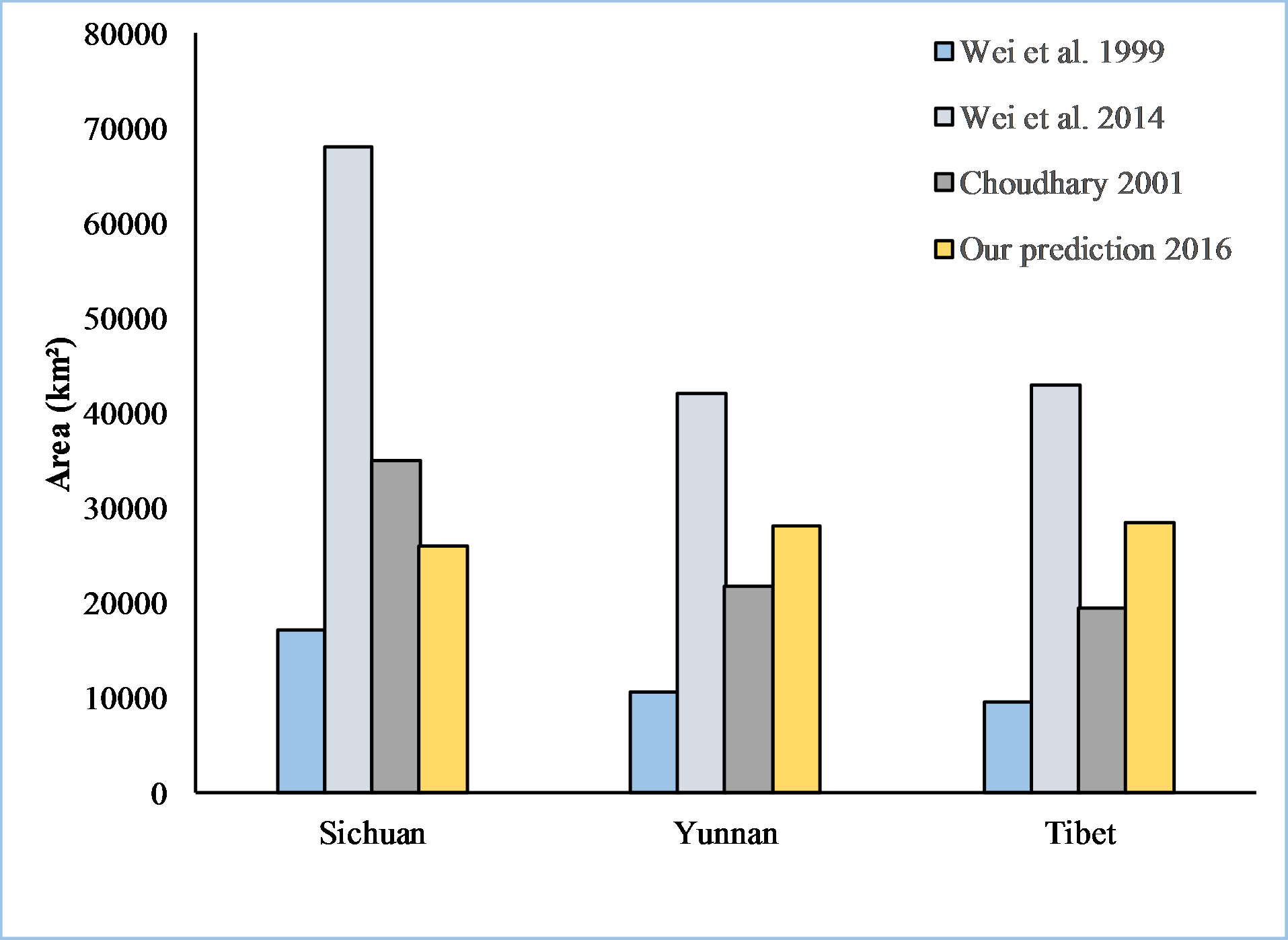


**Fig. S7.** Area of red panda habitat estimated in three provinces of China.


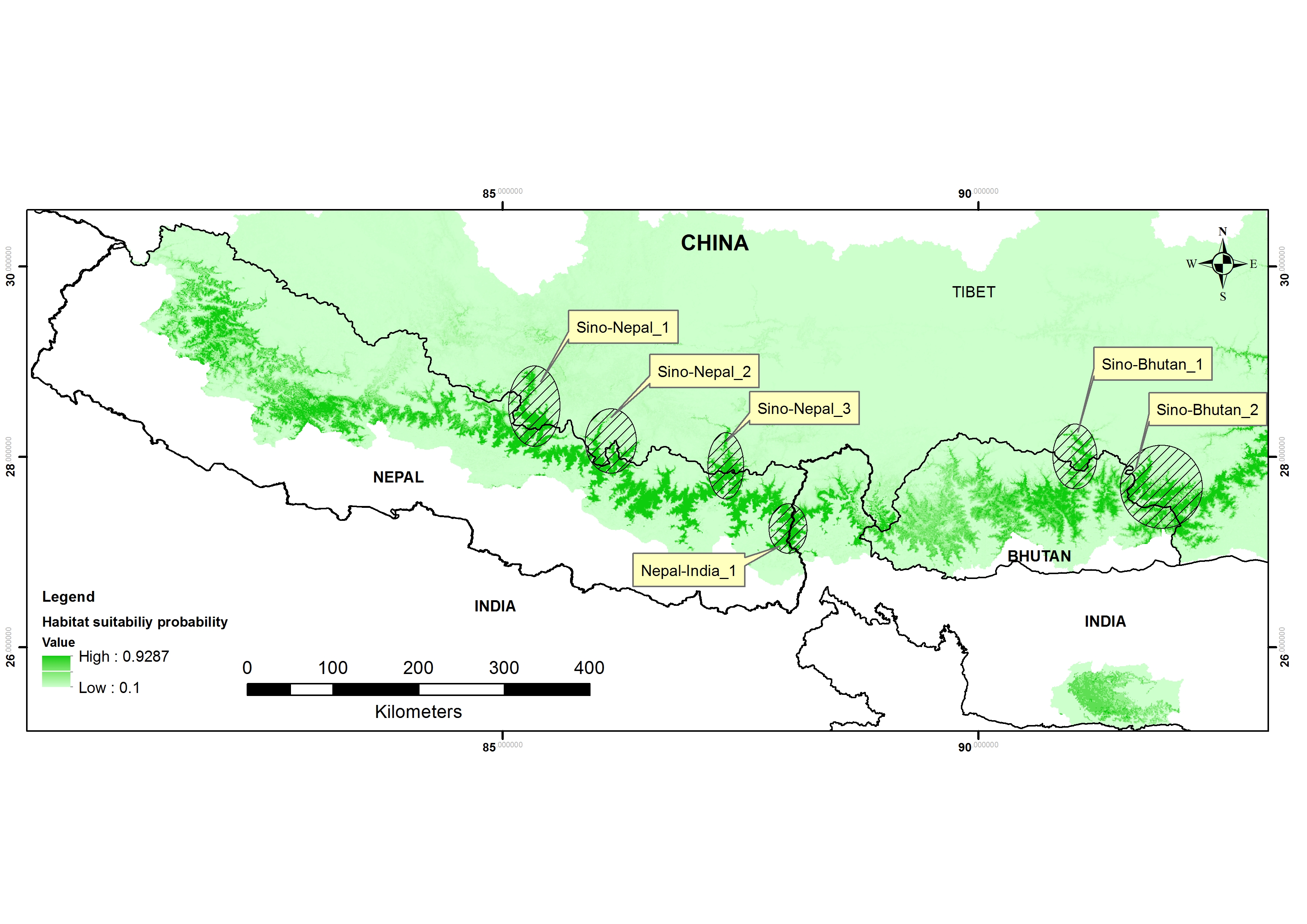


Fig. S8. Transboundary red panda habitat in the Himalayas.


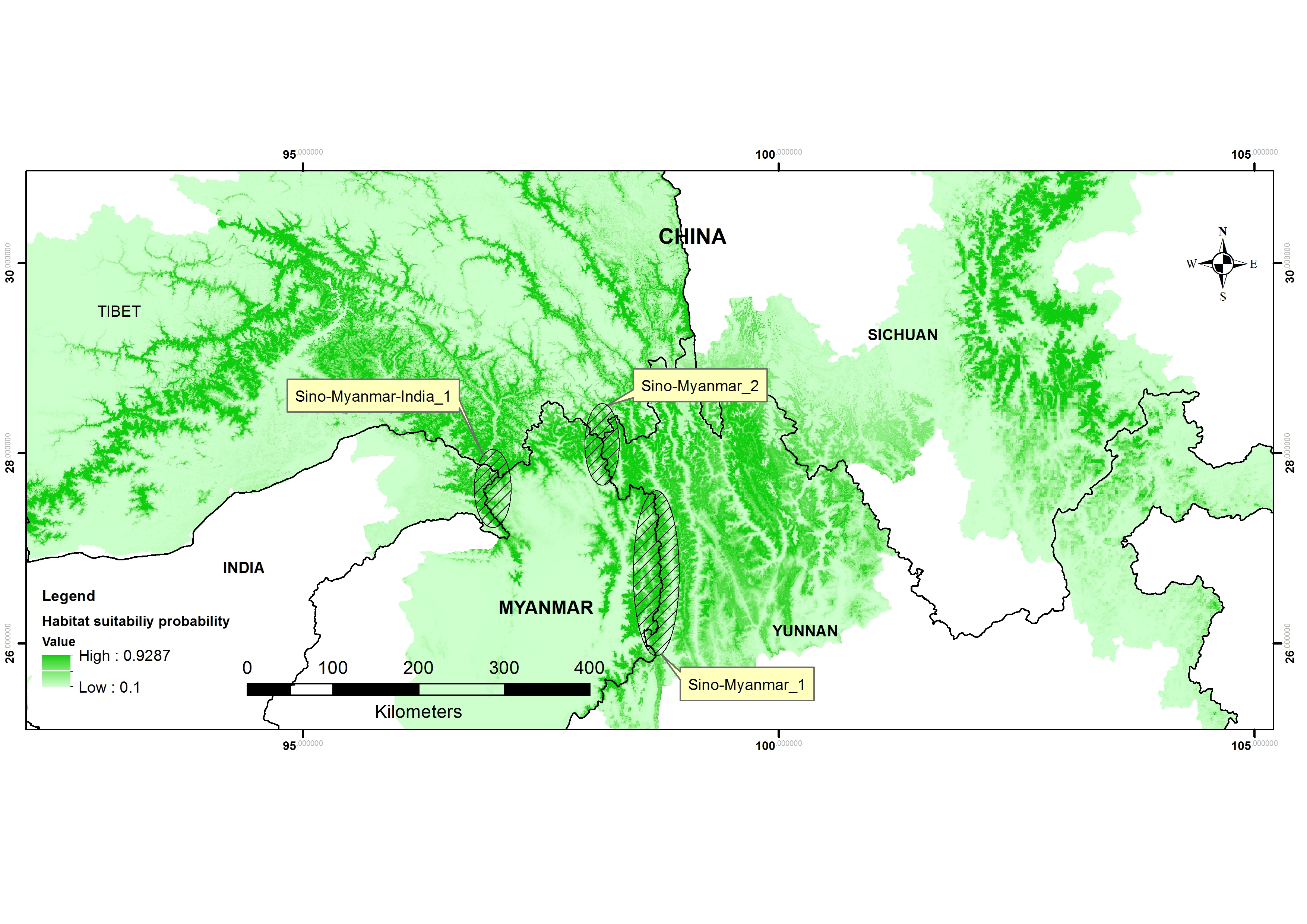


Fig. S9. Transboundary red panda habitat in Myanmar, Yunnan, Tibet and India.


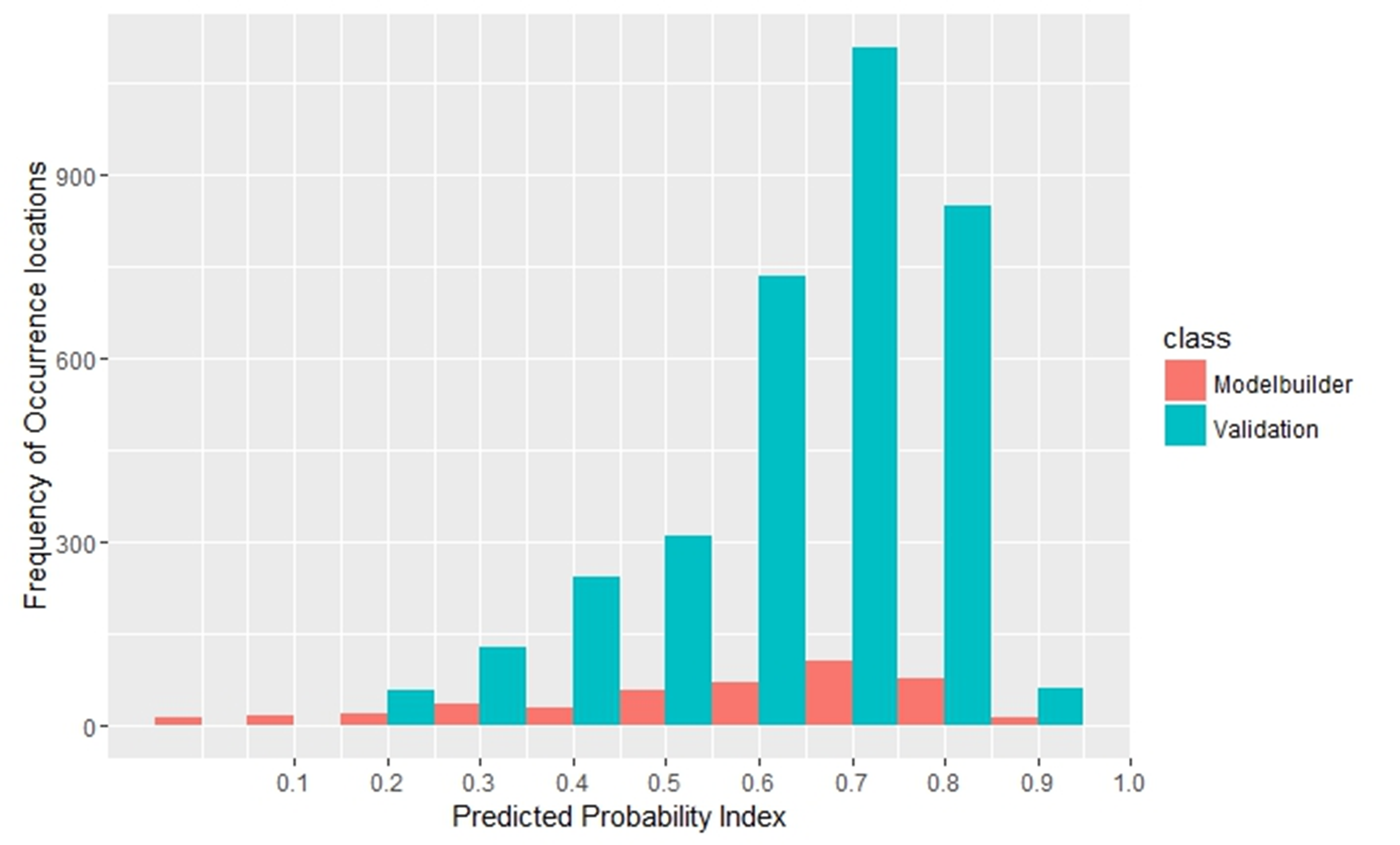


Fig. S 10. Frequency histogram of predictive vs actual red panda occurrence (databases for China and Nepal).


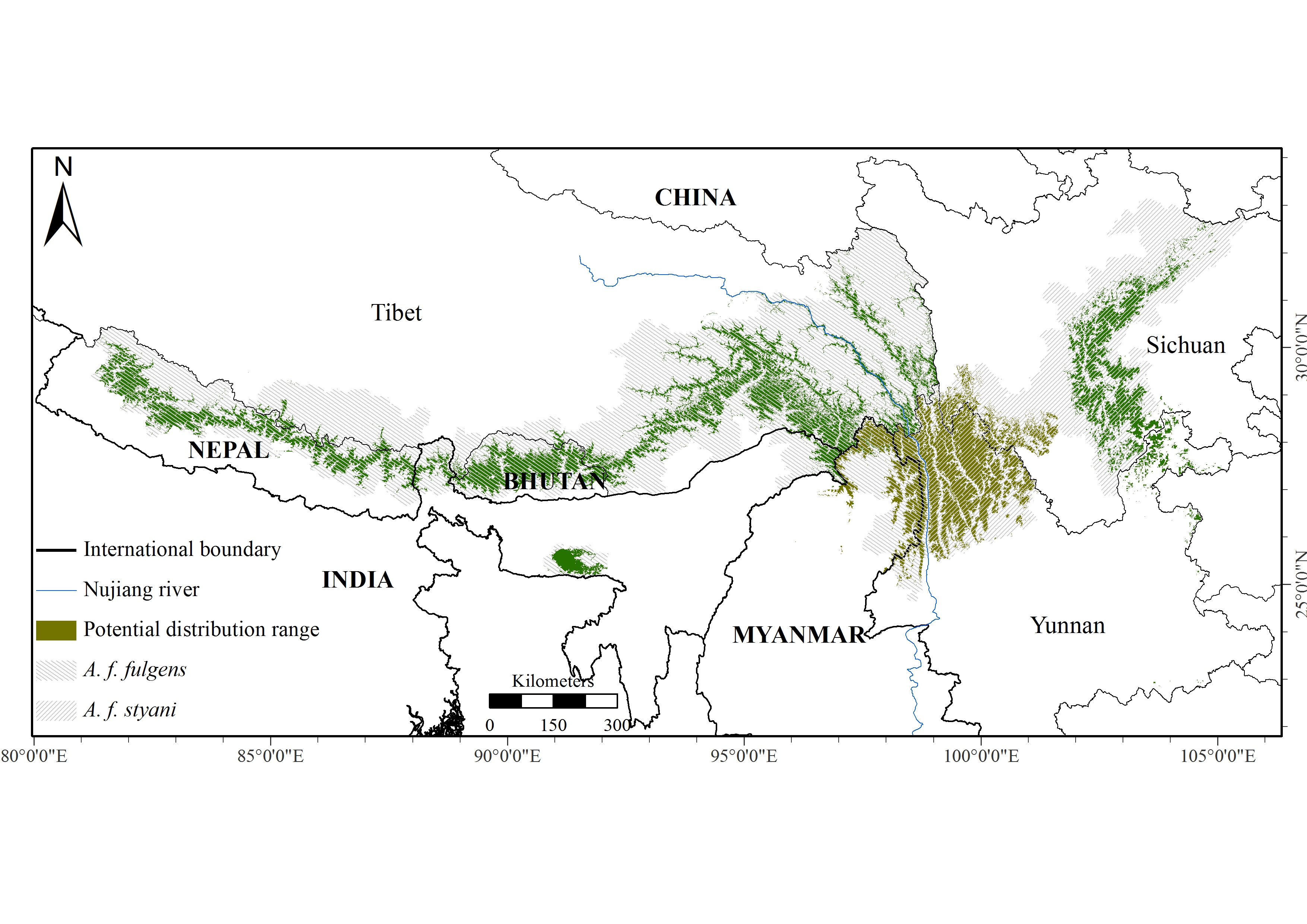


Fig.S11. Distribution of potential suitable habitat of red panda (Dark green color) representing both sub-species (inclined grey lines).
